# Evaluating the feasibility of using candidate DNA barcodes in discriminating species of the large Asteraceae family

**DOI:** 10.1186/1471-2148-10-324

**Published:** 2010-10-26

**Authors:** Ting Gao, Hui Yao, Jingyuan Song, Yingjie Zhu, Chang Liu, Shilin Chen

**Affiliations:** 1 Institute of Medicinal Plant Development, Chinese Academy of Medical Sciences, Peking Union Medical College, Beijing, PR China; 2 College of Life Sciences, Qingdao Agricultural University, Qingdao, PR China

## Abstract

**Background:**

Five DNA regions, namely, *rbcL*, *matK*, ITS, ITS2, and *psbA-trnH*, have been recommended as primary DNA barcodes for plants. Studies evaluating these regions for species identification in the large plant taxon, which includes a large number of closely related species, have rarely been reported.

**Results:**

The feasibility of using the five proposed DNA regions was tested for discriminating plant species within Asteraceae, the largest family of flowering plants. Among these markers, ITS2 was the most useful in terms of universality, sequence variation, and identification capability in the Asteraceae family. The species discriminating power of ITS2 was also explored in a large pool of 3,490 Asteraceae sequences that represent 2,315 species belonging to 494 different genera. The result shows that ITS2 correctly identified 76.4% and 97.4% of plant samples at the species and genus levels, respectively. In addition, ITS2 displayed a variable ability to discriminate related species within different genera.

**Conclusions:**

ITS2 is the best DNA barcode for the Asteraceae family. This approach significantly broadens the application of DNA barcoding to resolve classification problems in the family Asteraceae at the genera and species levels.

## Background

Asteraceae is the largest family of flowering plants in the world. The family includes over 1,600 genera and 23,000 individual species. Many members of the Asteraceae family are important for medicinal, ornamental, and economic purposes.

Approximately 300 Asteraceae species are already used for medicinal purposes in China. For example, *Artemisia annua *and its derivatives are effective in treating malaria [1]. *Saussurea involucrate*, an endangered species, possesses anti-fatigue, anti-inflammation, anti-tumor and free radical scavenging properties [2]. *Echinacea *also has immuno-modulatory properties with its ability to reduce inflammation, speed up wound healing and boost the immune system in response to bacterial or viral infection [3]. Commercially important plants of the Asteraceae family include the food crops *Lactuca sativa *(lettuce), *Cichorium intybus *(chicory), *Cynara scolymus *(globe artichoke), *Smallanthus sonchifolius *(yacon), *Helianthus tuberosus *(jerusalem artichoke), and so on. Aside from consumption, the seeds of *Helianthus annuus *(sunflower), and those of *Carthamus tinctorius *(safflower), another Asteraceae member, can be used for the production of cooking oil. Other commercially important species of the family Asteraceae are members of the *Tanacetum*, *Chrysanthemum *and *Pulicaria *genera, which have insecticidal properties. *Eupatorium adenophorum *is also one of the more noxious invasive plants worldwide, and it does have a significant effect on local ecosystems.

The wide variety of plants in the family Asteraceae often makes identification at the species level difficult [4]. Given the many valuable members of Asteraceae described above, an easy and accurate method of authenticating an Asteraceae species is indispensable for ensuring the drug and food safety of internationally traded herbs.

DNA barcoding is a process that uses a short piece of DNA sequence from a standard locus as a species identification tool [5]. DNA barcode regions have already been adopted for animal use [6,7] and several regions have previously been recommended for plant use [8-17]. The Plant Working Group of the Consortium for the Barcode of Life (CBOL) proposed *rbcL *and *matK *as the core DNA barcodes for plants [18]. A previous study by Kress et al. [9] tested 7 promising barcodes. However, only 15 sequences of Asteraceae, representing 14 species distributed among only 9 genera, were analyzed in the study. The CBOL Plant Working Group [18] also evaluated the performance of the leading barcoding loci in species identification, but the sequences of Asteraceae used included just 75 samples, consisting of 38 species belonging to 19 genera. Chen et al. [12] likewise compared the practicality of using the suggested barcode sequences against a large number of medicinal plants. However, the study included no more than 450 sequences of Asteraceae derived from 306 species from 50 genera. The researchers did not provide sufficient evidence that the recommended DNA barcode regions are suitable for species identification in the family Asteraceae, which includes a large number of closely related species. Thus, this issue is addressed in our study by comparing the feasibility of using each of these five proposed DNA barcodes (*rbcL*, *matK*, ITS, ITS2, and *psbA-trnH*) in the Asteraceae family.

## Results and Discussion

### Assessment of the universality of the five candidate barcodes

**Figure 1:**
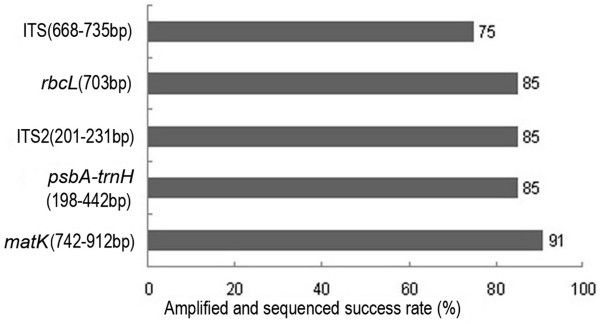
**Assessment of the universality of the five candidate barcodes**.

A universal DNA barcode is required to be tractable for use in a wide range of species. Therefore barcode regions must be relatively short in length to facilitate DNA extraction, amplification and sequencing [9]. As shown in Figure 1, for the selected samples, three regions (ITS2, *psbA-trnH *and *rbcL*) were amplified using a single pair of universal primers for each locus that results in high amplification and a sequencing efficiency of 85%. In comparison, ITS had a relatively lower efficiency at 75%. We used two pairs of *matK *primers exhibiting different universalities for the members of the family Asteraceae. The primers Kim3F/1R and 390F/1326R achieved amplifying and sequencing efficiencies of 91% and 25%, respectively.

### Measurement of inter- versus intra-specific genetic divergence at each locus

**Figure 2:**
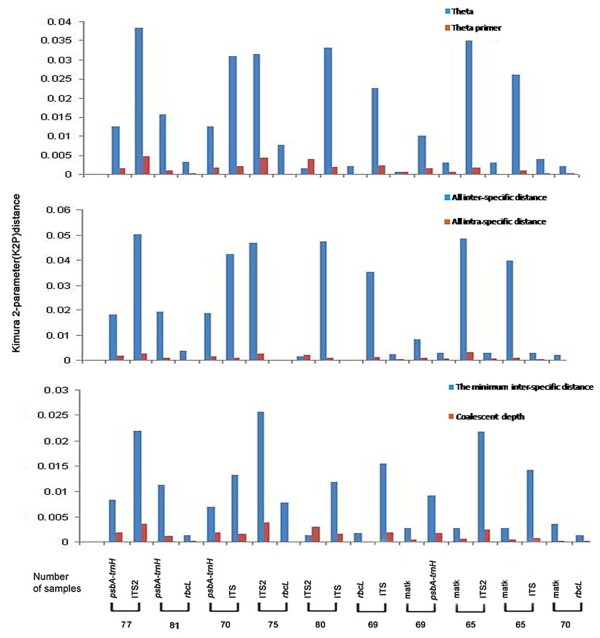
**Analyses of the inter-specific divergence between congeneric species and intra-specific variation of the five loci**. First, three parameters were used to characterize inter-specific divergence: (i) average inter-specific distance (K2P distance) between all species in each genus with at least two species; (ii) average theta prime (θ'), where theta prime is the mean pairwise distance within each genus with more than one species, thus eliminating biases associated with different numbers of species among genera; and (iii) smallest inter-specific distance, i.e., the minimum inter-specific distance within each genus with at least two species. Second, three additional parameters were used to determine intra-specific variation: (i) average intra-specific difference (K2P distance), that between all samples collected within each species with more than one individual; (ii) theta (θ), where theta is the mean pairwise distance within each species with at least two representatives; θ eliminates biases associated with unequal sampling among a species; and (iii) average coalescent depth, which is the maximum intra-specific distance within each species with at least two individuals.

Six metrics were employed to characterize inter- versus intra-specific variation (Figure 2) [11,12,19-21]. A favorable barcode should possess a high inter-specific divergence to distinguish different species. ITS2 and ITS both exhibited significantly higher levels of inter-specific discriminatory ability than *psbA-trnH *and *matK*. The lowest divergence between conspecific individuals, as determined by all inter-specific calculations was exhibited by *rbcL*. Wilcoxon signed-rank tests affirmed that ITS2 had the highest divergence at the inter-specific level, whereas *rbcL *had the lowest (Table 1). The results of the intra-specific differences were similar, with ITS2 contributing the largest and *rbcL *the smallest variations (Figure 2).

**Table 1:** Wilcoxon signed rank test of the inter-specific divergences among the five loci

W +	W-	inter Relative Ranks, n, *P *value	Result
ITS2	*psbA-trnH*	W + = 92, W - = 13, n = 14, *P *< 0.0132	ITS2 >*psbA-trnH*
*psbA-trnH*	*rbcL*	W + = 89, W - = 2, n = 13, *P *< 0.0024	*psbA-trnH > rbcL*
ITS	*psbA-trnH*	W + = 65, W - = 13, n = 12, *P *< 0.0414	ITS >*psbA-trnH*
*psbA-trnH*	*matK*	W + = 21, W - = 0, n = 6, *P *< 0.0277	*psbA-trnH > matK*
ITS2	*rbcL*	W + = 36, W - = 0, n = 8, *P *< 0.0117	ITS2 >*rbcL*
ITS2	ITS	W + = 355, W - = 23, n = 27, *P *< 6.6389 × 10^-5^	ITS2 > ITS
ITS2	*matK*	W + = 45, W - = 0, n = 9, *P *< 0.0076	ITS2*>matK*
ITS	*rbcL*	W + = 45, W - = 0, n = 9, *P *< 0.0076	ITS*>rbcL*
*rbcL*	*matK*	W + = 3, W - = 7, n = 4, *P *< 0.4615	*rbcL = matK*
ITS	*matK*	W + = 45, W - = 0, n = 9, *P *< 0.0075	ITS*>matK*

### Testing the efficacy of authentication

**Figure 3:**
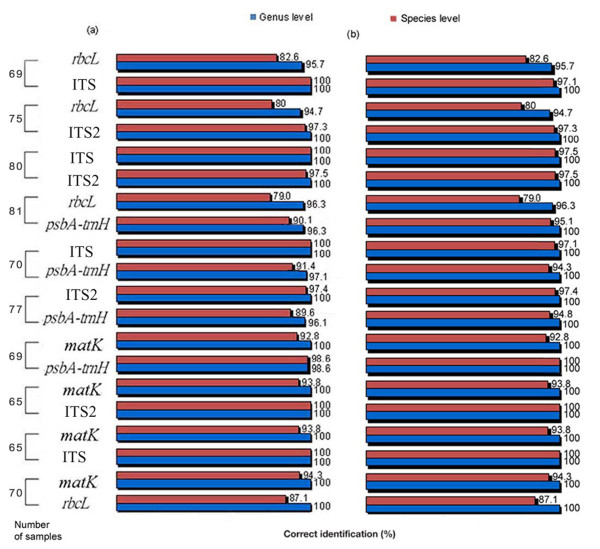
**Comparison of authentication efficiency of the five loci using two methods (a) BLAST1 method, and (b) Distance method**.

BLAST1 and Distance methods were used to test the ability of the potential barcoding sequences in assigning unique species identities to the given samples [12,22]. The results from the two methods revealed a clear pattern (Figure 3), demonstrating that the ITS region exhibits the highest identification efficiency. ITS2 and ITS performed well at the genus level using both methods, and at the species level using the Distance method. Using the BLAST1 method, ITS2 was less efficient (2.5%) than ITS at the species level, while *rbcL *was the lowest performer. In addition, except for the combination of *matK *and *psbA-trnH*, which improved the correct identification rates by 1.4%, using one sequence rather than a combination of two markers didn't improve the rates of identification.

The meta-analysis of markers, ITS, *psbA-trnH*, *matK *and *rbcL*, was also performed in parallel with the analysis on ITS2 using GenBank data (see Additional file 1: Identification efficiency of the five regions evaluated in a large pool of Asteraceae samples from GenBank). The correct identification rates were significantly higher for ITS2 than for other markers except ITS. The GenBank data analyses were consistent with our experimental data results. Compared with single markers alone, combinations of markers could improve the rate of correct species identification (<5%).

Overall, our study demonstrates that ITS2 is the most successful region in terms of universality, the specific genetic divergence, and discrimination between species among the five markers examined. ITS is also proven as a valuable marker for authenticating species in Asteraceae. However, its low amplification efficiency limits its potential for broad taxonomic use. Although *matK*, *rbcL*, and *psbA-trnH *have effective primers for the amplification, the three markers are less powerful than ITS and ITS2 in species discrimination in the family Asteraceae. Moreover, theoretically, the regions based on nuclear DNA are much more informative than barcodes based on organellar DNA [23].

To evaluate further the ability of the ITS2 region to authenticate a wide range of Asteraceae species, it was also tested against a larger database that includes 3,490 samples sequences derived from 2,315 different species (Table 2). The ITS2 region performed well, with a 76.4% (BLAST1 method) and 69.7% (Distance method) successful identification rate at the species level and a 97.4% (BLAST1 method) and 96.2% (Distance method) successful identification rate at the genus level.

**Table 2:** Identification efficiency of the ITS2 locus for the family and six large genera in dataset 2 using different methods

Category		Method	No. of samples	No. of species	Success identification (%) at the species level/ at the genus level
Genus	*Centaurea*	BLAST1	204	157	48.0/-
		Distance	204	157	45.6/-
	*Senecio*	BLAST1	203	157	73.4/-
		Distance	203	157	70.9/-
	*Artemisia*	BLAST1	91	74	59.3/-
		Distance	91	74	51.6/-
	*Stevia*	BLAST1	91	75	76.9/-
		Distance	91	75	71.4/-
	*Erigeron*	BLAST1	87	65	80.5/-
		Distance	87	65	75.9/-
	*Brachyscome*	BLAST1	57	55	96.5/-
		Distance	57	55	96.5/-
Family	Asteraceae	BLAST1	3,490	2,315	76.4/97.4
		Distance	3,490	2,315	69.4/96.2

### ITS2 is suitable, but not ideal

Our research displayed a similar trend to that of Chen et al. [12] and demonstrated that ITS2 is a promising barcode for authenticating plant species. In accordance with the criteria outlined by Kress et al. [9], the ITS2 region has several advantages that make it a promising candidate for DNA barcoding. It has been proposed as a candidate marker for taxonomic classification and barcoding of medicinal plants because it has both high correct identification rate and high amplification efficiency [12,24-27]. As the ITS2 region is one of the most common regions used for phylogenetic analyses [28-30], a vast amount of sequencing data has already been deposited in GenBank and is ready for immediate use.

The presence of multiple copies of ITS2 sequences is challenging [13]. However, Coleman [27] proposed that the repeats displayed a high degree of similarity. Coleman also suggested that the PCR-amplified copies could represent the information of the ITS2 region in individuals and that ITS2 could be considered a single locus in most cases.

Among the six large genera (number of species > 50) in the Asteraceae family (Table 2), the utility of ITS2 for species authentication varied and could only be analyzed individually, not as a group. For the genus *Brachyscome*, with 57 sequences representing 55 species, ITS2 worked well with a 96.5% successful identification rate. Satisfactory results were also obtained for the genus *Erigeron*, where >80.5% of the sequences were correctly identified. In contrast, ITS2 had lower identification efficiency for the genera *Centaurea *and *Artemisia *(48.0% and 59.3%, respectively). And in two other genera (*Senecio *and *Stevia*), ITS2 was relatively powerful for taxonomic classification, precisely authenticating 73.4% and 76.9% of the samples, respectively. The identification efficiencies of ITS2 in dataset 2 are listed in Additional file 2 (Authentication efficiency of ITS2 using different methods for the genera in dataset 2 containing more than one species).

To improve identification accuracy within a particular genus, using combinations of DNA barcodes may be necessary. Therefore, ITS is proposed for use as a complementary barcode for differentiating species within the Asteraceae family.

### Application and meaning of DNA barcoding

The selected DNA barcode for Asteraceae, ITS2, is not perfect, especially for taxonomists and phylogenetic experts. However, even an imperfect barcode can have a major effect on many areas of research and be sufficient for many applications [13]. For instance, ITS2 might be a suitable DNA barcode for public users, such as customs officials, forensic examiners, food-processing individuals, and research organizations. Considering that ITS2 has a strong ability to group plant samples into their correct genus and has a relatively high accuracy for grouping samples into their correct species, it is of great practical value to individuals without adequate taxonomic training. Compared with ITS2, ITS or the chloroplast genome is better equipped to deal with the biological complexities of species distinctions, a major focus of taxonomists and phylogenetic experts [13].

## Conclusions

Altogether, our results support the claim that ITS2 is a valuable locus for differentiating species within Asteraceae and that DNA barcoding is a useful tool for classification and identification of individual species. We propose applying DNA barcoding technology to resolve classification problems in the family Asteraceae at the genera and species levels.

## Methods

### Sampling of plant materials

Dataset 1, which consists of 110 samples from 63 species representing 48 genera of Asteraceae (see Additional file 3: Samples in dataset 1 for testing the potential barcodes and accession numbers in GenBank) was gathered from a large geographical area in China from July 2007 to January 2008. Great effort was made to ensure that the samples represent the major lineages of Asteraceae. Furthermore, the maximum number of samples belonging to closely related species was collected (Table 3). Plant samples in dataset 1 were spread across two subfamilies (Carduoideae and Cichorioideae) and encompassed a total of 11 tribes of the family Asteraceae.

**Table 3:** Number of DNA sequences used in the study

Dataset	Markers	Total No. of sequences	No. of sequences belonging to genera containing more than one species	No. of sequences belonging to species containing more than one samples
Dataset 1	*psbA-trnH*	93(56)	72(34)	59(21)
	ITS2	93(58)	70(35)	55(20)
Dataset 2	ITS	83(55)	60(32)	48(20)
	*rbcL*	93(57)	68(32)	56(20)
	*matK*	80(47)	20(11)	53(20)
	ITS2	3,490(2,315)	2,973(1,877)	1,748(583)

The numbers of species to which these sequences belong are shown in parentheses.

### DNA extraction, PCR amplification, and sequencing

DNA extraction, PCR amplification, and sequencing were performed as described previously [12].

### Data Acquisition from GenBank

First, all sequences involving the five markers of Asteraceae were downloaded from GenBank. Certain gene regions of the five barcoding markers based on GenBank annotations were then obtained. Sequences <100 bp in length, with ambiguous bases (more than 15'Ns'), or those belonging to unnamed species (i.e. sequences with 'sp.' in the species name) were filtered out. Finally, to avoid contamination with fungal sequences existing in ITS2 sequences, a Hidden Markov Model (HMM) [31] based on well-curated fungal sequences was used to search for downloaded ITS2 sequences to remove the sequences possibly contaminated with fungi. The meta-analysis was performed using the remaining sequences (see Additional file 4: Accession numbers of the five loci sequences from GenBank for the meta-analysis). The ITS2 sequences were also used to construct dataset 2, which is comprised of 3,490 sequences from 2,315 Asteraceae species downloaded from GenBank (see Additional file 5: Accession numbers of ITS2 sequences used in dataset 2). Many closely related species were also included in dataset 2 (Table 3).

### Sequence alignment and analysis

Consensus sequences and contig generation were accomplished using CodonCode Aligner V 3.5 (CodonCode Co., USA). The sequences of the candidate DNA barcodes were aligned using Clustal W and the genetic distances were calculated using the Kimura 2-Parameter (K2P) model. The average intra-specific distance, theta, and coalescent depth were calculated to evaluate the intra-specific variation [12,19]-[21]. The average inter-specific distance, the minimum inter-specific distance, and Theta primer were used to represent inter-specific divergences [11,12,20,21]. Wilcoxon signed-rank tests were used as previously described [10-12]. Two methods of species identification, namely BLAST1 and the nearest distance method, were performed as described previously [12,22]. The traffic light approach [32] was used to identify the combination of markers, as long as the sequences could be identified by one of the markers in combination, the combination would have identification power. If any of the sequences were identified unsuccessfully for any marker in combination, the combination would incapable of identifying that sequence.

## Authors' contributions

TG, JYS, SLC, CL conceived of the project and designed experiments. TG and HY performed the experiments. TG and YJZ analyzed the data. TG, HY wrote the paper. All authors read and approved the final manuscript.

## Supplementary Material

Additional file 1 **Identification efficiency of the five regions evaluated in a large pool of Asteraceae samples from GenBank**. For each marker and marker combination, number of samples used for identification and the correct identification rates at the species and genus levels are shown. Click here for file

Additional file 2 **Authentication efficiency of ITS2 using different methods for the genera in dataset 2 containing more than one species**. For each genus, number of samples and species used for identification and the correct identification rates using different methods are shown. Click here for file

Additional file 3 **Samples in dataset 1 for testing the potential barcodes and accession numbers in GenBank**. For each samples in dataset 1, the latin name and accession numbers in GenBank are shown. Click here for file

Additional file 4 **Accession numbers of the five loci sequences from GenBank for the meta-analysis**. For each samples used for the meta-analysis, the accession numbers in GenBank are shown. Click here for file

Additional file 5 **Accession numbers of ITS2 sequences used in dataset 2**. For each samples used in dataset 2, the accession numbers in GenBank are shown. Click here for file
