# Supplementary material for: Evaluating the feasibility of using candidate DNA barcodes in discriminating species of the large Asteraceae family
Source: BMC Evol Biol. 2010 Oct 26;10:324. doi: 10.1186/1471-2148-10-324 (PMC3087544; doi:10.1186/1471-2148-10-324)
Supplement: Additional file 1 — Identification efficiency of the five regions evaluated in a large pool of Asteraceae samples from GenBank. For each marker and marker combination, number of samples used for identification and the correct identification rates at the species and genus levels are shown. [file 1471-2148-10-324-S1.DOC]

### Additional file 1 –Identification efficiency of the five regions evaluated in a large pool of Asteraceae samples from GenBank

| Markers | | No. of  samples | Correct identification% | |
| --- | --- | --- | --- | --- |
|  |  |  | Genus level | Species level |
| ITS2-*psbA-trnH* | ITS2 | 583 | 95.9 | 84.7 |
|  | *psbA-trnH* | 583 | 82.3 | 52.5 |
|  | ITS2+ *psbA-trnH* | 583 | 98.1 | 89.0 |
| ITS2-*matk* | ITS2 | 158 | 100 | 96.2 |
|  | *matk* | 158 | 88.6 | 66.5 |
|  | ITS2+*matk* | 158 | 100 | 96.8 |
| ITS2-*rbcL* | ITS2 | 126 | 100 | 98.4 |
|  | *rbcL* | 126 | 100 | 92.9 |
|  | ITS2+ *rbcL* | 126 | 100 | 100 |
| ITS2-ITS | ITS2 | 700 | 97.0 | 87.0 |
|  | ITS | 700 | 98.9 | 95.3 |
|  | ITS2+ITS | 700 | 98.9 | 95.3 |
| *matk*-*rbcL* | *matk* | 210 | 99.5 | 82.9 |
|  | *rbcL* | 210 | 99.0 | 75.2 |
|  | *matk* +*rbcL* | 210 | 100 | 86.2 |
| ITS2-*matk*-*rbcL* | ITS2 | 78 | 100 | 98.7 |
|  | *matk* | 78 | 98.7 | 85.9 |
|  | *rbcL* | 78 | 100 | 91.0 |
|  | *matk* + *rbcL* | 78 | 100 | 94.9 |
