# Supplementary material for: Evaluating the feasibility of using candidate DNA barcodes in discriminating species of the large Asteraceae family
Source: BMC Evol Biol. 2010 Oct 26;10:324. doi: 10.1186/1471-2148-10-324 (PMC3087544; doi:10.1186/1471-2148-10-324)
Supplement: Additional file 2 — Authentication efficiency of ITS2 using different methods for the genera in dataset 2 containing more than one species. For each genus, number of samples and species used for identification and the correct identification rates using different methods are shown. [file 1471-2148-10-324-S2.DOC]

**Additional file 2 –Authentication efficiency of ITS2 using different methods for the genera in dataset 2 containing more than one species**

| Genus | No.of species | No.of samples | Correct identification（%） | | |
| --- | --- | --- | --- | --- | --- |
|  |  |  | Blast 1 | | distance |
|  |  |  | at genus level | at species level | at species level |
| *Centaurea* | 157 | 204 | 99.0 | 48.0 | 45.6 |
| *Senecio* | 157 | 203 | 99.5 | 73.4 | 70.9 |
| *Artemisia* | 75 | 91 | 100 | 59.3 | 51.6 |
| *Stevia* | 75 | 91 | 100 | 76.9 | 71.4 |
| *Erigeron* | 65 | 87 | 100 | 80.5 | 75.9 |
| *Brachyscome* | 55 | 57 | 100 | 96.5 | 96.5 |
| *Helianthus* | 46 | 69 | 100 | 52.2 | 34.8 |
| *Chaetanthera* | 44 | 80 | 100 | 88.8 | 76.3 |
| *Saussurea* | 44 | 54 | 100 | 98.1 | 94.4 |
| *Euryops* | 43 | 75 | 100 | 78.7 | 60.0 |
| *Melampodium* | 41 | 115 | 100 | 85.2 | 79.1 |
| *Dahlia* | 34 | 50 | 74.0 | 50.0 | 40.0 |
| *Symphyotrichum* | 33 | 64 | 100 | 46.9 | 46.9 |
| *Taraxacum* | 28 | 210 | 100 | 72.9 | 66.7 |
| *Chrysanthemum* | 26 | 56 | 39.3 | 30.4 | 23.2 |
| *Bidens* | 26 | 34 | 100 | 64.7 | 50.0 |
| *Ligularia* | 25 | 33 | 100 | 93.9 | 93.9 |
| *Jacobaea* | 24 | 28 | 100 | 92.9 | 82.1 |
| *Cineraria* | 21 | 26 | 100 | 50.0 | 50.0 |
| *Blumea* | 21 | 24 | 100 | 83.3 | 79.2 |
| *Berkheya* | 21 | 23 | 100 | 91.3 | 91.3 |
| *Flaveria* | 20 | 68 | 100 | 86.8 | 86.8 |
| *Leontopodium* | 20 | 40 | 100 | 57.5 | 35.0 |
| *Vernonia* | 20 | 23 | 82.6 | 65.2 | 60.9 |
| *Abrotanella* | 19 | 23 | 100 | 78.3 | 78.3 |
| *Lasthenia* | 18 | 56 | 100 | 87.5 | 82.1 |
| *Gaillardia* | 18 | 41 | 100 | 53.7 | 51.2 |
| *Rhaponticum* | 18 | 21 | 100 | 95.2 | 90.5 |
| *Dasyphyllum* | 18 | 20 | 100 | 80.0 | 55.0 |
| *Arnica* | 17 | 39 | 100 | 87.2 | 84.6 |
| *Hypochaeris* | 17 | 36 | 100 | 80.6 | 77.8 |
| *Dubautia* | 17 | 26 | 100 | 42.3 | 42.3 |
| *Calotis* | 17 | 18 | 100 | 100 | 100 |
| *Gazania* | 16 | 40 | 100 | 52.5 | 47.5 |
| *Encelia* | 16 | 27 | 100 | 55.6 | 44.4 |
| *Brachyglottis* | 16 | 20 | 100 | 100 | 100 |
| *Hieracium* | 15 | 27 | 100 | 59.3 | 59.3 |
| *Munnozia* | 15 | 20 | 100 | 100 | 100 |
| *Arctotis* | 14 | 16 | 100 | 75.0 | 75.0 |
| *Lessingia* | 13 | 30 | 100 | 26.7 | 26.7 |
| *Scorzoneroides* | 13 | 17 | 100 | 100 | 88.2 |
| *Smallanthus* | 13 | 14 | 100 | 57.1 | 42.9 |
| *Scorzonera* | 13 | 13 | 100 | 69.2 | 69.2 |
| *Leontodon* | 12 | 18 | 100 | 72.2 | 55.6 |
| *Microseris* | 12 | 16 | 100 | 37.5 | 37.5 |
| *Bellis* | 11 | 23 | 100 | 87.0 | 39.1 |
| *Ajania* | 11 | 14 | 28.6 | 28.6 | 35.7 |
| *Olearia* | 11 | 12 | 100 | 75.0 | 66.7 |
| *Chuquiraga* | 10 | 19 | 100 | 84.2 | 68.4 |
| *Barnadesia* | 9 | 11 | 100 | 90.9 | 81.8 |
| *Othonna* | 9 | 11 | 100 | 81.8 | 81.8 |
| *Osteospermum* | 9 | 9 | 100 | 66.7 | 66.7 |
| *Celmisia* | 8 | 8 | 100 | 100 | 62.5 |
| *Conyza* | 8 | 8 | 100 | 100 | 100 |
| *Thelesperma* | 7 | 17 | 100 | 70.6 | 70.6 |
| *Coreocarpus* | 7 | 13 | 100 | 92.3 | 61.5 |
| *Ichthyothere* | 7 | 7 | 100 | 57.1 | 42.9 |
| *Klasea* | 7 | 7 | 100 | 100 | 71.4 |
| *Cynara* | 6 | 20 | 100 | 95.0 | 70.0 |
| *Haplocarpha* | 6 | 10 | 100 | 100 | 100 |
| *Cichorium* | 6 | 6 | 100 | 50.0 | 50.0 |
| *Hinterhubera* | 6 | 6 | 100 | 100 | 100 |
| *Monticalia* | 6 | 6 | 100 | 66.7 | 66.7 |
| *Ozothamnus* | 6 | 6 | 100 | 100 | 100 |
| *Elephantopus* | 5 | 9 | 100 | 100 | 100 |
| *Parastrephia* | 5 | 8 | 100 | 62.5 | 62.5 |
| *Picris* | 5 | 6 | 100 | 100 | 66.7 |
| *Felicia* | 5 | 5 | 100 | 100 | 100 |
| *Lepidaploa* | 5 | 5 | 100 | 60.0 | 60.0 |
| *Mutisia* | 5 | 5 | 100 | 100 | 100 |
| *Sinclairia* | 5 | 5 | 100 | 100 | 100 |
| *Pluchea* | 4 | 21 | 90.5 | 38.1 | 47.6 |
| *Doellingeria* | 4 | 15 | 100 | 86.7 | 6.7 |
| *Leucogenes* | 4 | 12 | 100 | 83.3 | 83.3 |
| *Tragopogon* | 4 | 12 | 100 | 66.7 | 66.7 |
| *Chromolaena* | 4 | 9 | 100 | 100 | 100 |
| *Emilia* | 4 | 8 | 100 | 100 | 100 |
| *Lagenophora* | 4 | 7 | 100 | 100 | 100 |
| *Gnaphalium* | 4 | 6 | 100 | 100 | 100 |
| *Pericallis* | 4 | 6 | 100 | 100 | 100 |
| *Aster* | 4 | 5 | 100 | 100 | 100 |
| *Cullumia* | 4 | 5 | 100 | 100 | 100 |
| *Enceliopsis* | 4 | 5 | 100 | 60.0 | 60.0 |
| *Hertia* | 4 | 5 | 100 | 40.0 | 40.0 |
| *Iranecio* | 4 | 5 | 100 | 100 | 100 |
| *Polyachyrus* | 4 | 5 | 100 | 0.0 | 0.0 |
| *Rumfordia* | 4 | 5 | 100 | 100 | 100 |
| *Tanacetum* | 4 | 5 | 100 | 60.0 | 60.0 |
| *Anaphalioides* | 4 | 4 | 100 | 100 | 0.0 |
| *Anaphalis* | 4 | 4 | 100 | 100 | 100 |
| *Bellium* | 4 | 4 | 100 | 50.0 | 50.0 |
| *Carduus* | 4 | 4 | 100 | 100 | 100 |
| *Chersodoma* | 4 | 4 | 100 | 100 | 100 |
| *Culcitium* | 4 | 4 | 100 | 75.0 | 50.0 |
| *Elekmania* | 4 | 4 | 100 | 100 | 100 |
| *Graphistylis* | 4 | 4 | 100 | 25.0 | 25.0 |
| *Gynoxys* | 4 | 4 | 100 | 50.0 | 25.0 |
| *Haplopappus* | 4 | 4 | 100 | 100 | 50.0 |
| *Hirpicium* | 4 | 4 | 100 | 100 | 100 |
| *Layia* | 4 | 4 | 75.0 | 75.0 | 100 |
| *Myopordon* | 4 | 4 | 100 | 100 | 100 |
| *Pentanema* | 4 | 4 | 100 | 100 | 100 |
| *Perezia* | 4 | 4 | 100 | 100 | 100 |
| *Tagetes* | 4 | 4 | 100 | 100 | 100 |
| *Westoniella* | 4 | 4 | 100 | 100 | 50.0 |
| *Inula* | 3 | 7 | 100 | 28.6 | 28.6 |
| *Sigesbeckia* | 3 | 7 | 100 | 100 | 100 |
| *Curio* | 3 | 6 | 66.7 | 66.7 | 100 |
| *Achillea* | 3 | 5 | 100 | 100 | 100 |
| *Argyroxiphium* | 3 | 5 | 100 | 0.0 | 0.0 |
| *Linzia* | 3 | 5 | 100 | 100 | 100 |
| *Acanthospermum* | 3 | 4 | 100 | 100 | 100 |
| *Cymbonotus* | 3 | 4 | 100 | 100 | 100 |
| *Duhaldea* | 3 | 4 | 100 | 100 | 100 |
| *Helichrysum* | 3 | 4 | 100 | 100 | 100 |
| *Herrickia* | 3 | 4 | 100 | 100 | 100 |
| *Laggera* | 3 | 4 | 75.0 | 75.0 | 100 |
| *Liabum* | 3 | 4 | 100 | 100 | 100 |
| *Oreostemma* | 3 | 4 | 100 | 100 | 100 |
| *Aetheolaena* | 3 | 3 | 100 | 100 | 100 |
| *Amellus* | 3 | 3 | 100 | 100 | 100 |
| *Archibaccharis* | 3 | 3 | 100 | 100 | 100 |
| *Baccharis* | 3 | 3 | 100 | 100 | 100 |
| *Balduina* | 3 | 3 | 100 | 100 | 100 |
| *Bethencourtia* | 3 | 3 | 100 | 66.7 | 0.0 |
| *Dendrophorbium* | 3 | 3 | 100 | 100 | 100 |
| *Eriophyllum* | 3 | 3 | 100 | 100 | 100 |
| *Haastia* | 3 | 3 | 100 | 100 | 100 |
| *Hazardia* | 3 | 3 | 100 | 100 | 100 |
| *Hubertia* | 3 | 3 | 100 | 100 | 33.3 |
| *Hymenoxys* | 3 | 3 | 100 | 33.3 | 33.3 |
| *Isocoma* | 3 | 3 | 100 | 100 | 33.3 |
| *Kleinia* | 3 | 3 | 100 | 100 | 100 |
| *Laestadia* | 3 | 3 | 100 | 100 | 100 |
| *Matricaria* | 3 | 3 | 100 | 33.3 | 33.3 |
| *Monolopia* | 3 | 3 | 100 | 100 | 100 |
| *Nassauvia* | 3 | 3 | 100 | 100 | 100 |
| *Packera* | 3 | 3 | 100 | 100 | 100 |
| *Pentacalia* | 3 | 3 | 100 | 100 | 100 |
| *Polymnia* | 3 | 3 | 100 | 100 | 100 |
| *Psathyrotes* | 3 | 3 | 100 | 100 | 100 |
| *Pseudogynoxys* | 3 | 3 | 100 | 100 | 100 |
| *Pyrrocoma* | 3 | 3 | 100 | 33.3 | 0.0 |
| *Raoulia* | 3 | 3 | 100 | 100 | 33.3 |
| *Ruilopezia* | 3 | 3 | 100 | 100 | 100 |
| *Vittadinia* | 3 | 3 | 100 | 100 | 100 |
| *Paranephelius* | 2 | 15 | 100 | 93.3 | 53.3 |
| *Ambrosia* | 2 | 9 | 100 | 100 | 100 |
| *Carthamus* | 2 | 6 | 100 | 100 | 100 |
| *Mikania* | 2 | 6 | 100 | 100 | 100 |
| *Chrysanthemoides* | 2 | 5 | 100 | 100 | 100 |
| *Dolomiaea* | 2 | 5 | 100 | 100 | 100 |
| *Erato* | 2 | 5 | 100 | 100 | 100 |
| *Aztecaster* | 2 | 4 | 100 | 100 | 100 |
| *Erechtites* | 2 | 4 | 100 | 100 | 100 |
| *Helminthotheca* | 2 | 4 | 100 | 100 | 100 |
| *Chiliotrichum* | 2 | 3 | 33.3 | 33.3 | 33.3 |
| *Cosmos* | 2 | 3 | 100 | 100 | 100 |
| *Crassocephalum* | 2 | 3 | 100 | 100 | 100 |
| *Dendrosenecio* | 2 | 3 | 100 | 100 | 100 |
| *Didelta* | 2 | 3 | 100 | 100 | 100 |
| *Leptinella* | 2 | 3 | 100 | 100 | 100 |
| *Madia* | 2 | 3 | 33.3 | 33.3 | 100 |
| *Nardophyllum* | 2 | 3 | 100 | 100 | 100 |
| *Nidorella* | 2 | 3 | 100 | 100 | 100 |
| *Philoglossa* | 2 | 3 | 100 | 100 | 100 |
| *Rhaponticoides* | 2 | 3 | 66.7 | 66.7 | 33.3 |
| *Xanthium* | 2 | 3 | 100 | 100 | 100 |
| *Ageratina* | 2 | 2 | 100 | 100 | 100 |
| *Agoseris* | 2 | 2 | 100 | 100 | 100 |
| *Antennaria* | 2 | 2 | 100 | 100 | 100 |
| *Anthemis* | 2 | 2 | 100 | 100 | 100 |
| *Arida* | 2 | 2 | 100 | 100 | 100 |
| *Arrhenechthites* | 2 | 2 | 100 | 100 | 100 |
| *Baccharoides* | 2 | 2 | 100 | 100 | 100 |
| *Bolandia* | 2 | 2 | 100 | 100 | 100 |
| *Bothriocline* | 2 | 2 | 100 | 100 | 100 |
| *Calycera* | 2 | 2 | 100 | 100 | 100 |
| *Carramboa* | 2 | 2 | 100 | 100 | 100 |
| *Chiliophyllum* | 2 | 2 | 50.0 | 50.0 | 100 |
| *Chrysolaena* | 2 | 2 | 100 | 100 | 100 |
| *Coespeletia* | 2 | 2 | 100 | 100 | 100 |
| *Cotula* | 2 | 2 | 100 | 100 | 100 |
| *Crepis* | 2 | 2 | 100 | 100 | 100 |
| *Critoniopsis* | 2 | 2 | 100 | 100 | 100 |
| *Dendranthema* | 2 | 2 | 0.0 | 0.0 | 0.0 |
| *Eriocephalus* | 2 | 2 | 100 | 100 | 100 |
| *Espeletia* | 2 | 2 | 100 | 100 | 100 |
| *Espeletiopsis* | 2 | 2 | 50.0 | 50.0 | 100 |
| *Ewartia* | 2 | 2 | 100 | 100 | 100 |
| *Geraea* | 2 | 2 | 100 | 100 | 100 |
| *Gorteria* | 2 | 2 | 100 | 100 | 100 |
| *Guardiola* | 2 | 2 | 100 | 100 | 100 |
| *Helenium* | 2 | 2 | 100 | 100 | 100 |
| *Hesperomannia* | 2 | 2 | 100 | 100 | 100 |
| *Hilliardiella* | 2 | 2 | 100 | 50.0 | 0.0 |
| *Hulsea* | 2 | 2 | 100 | 100 | 100 |
| *Lagophylla* | 2 | 2 | 100 | 100 | 100 |
| *Leucheria* | 2 | 2 | 100 | 100 | 100 |
| *Leuzea* | 2 | 2 | 100 | 100 | 100 |
| *Llerasia* | 2 | 2 | 100 | 100 | 100 |
| *Madagaster* | 2 | 2 | 100 | 100 | 100 |
| *Marshallia* | 2 | 2 | 100 | 100 | 100 |
| *Minuria* | 2 | 2 | 100 | 100 | 100 |
| *Oritrophium* | 2 | 2 | 100 | 100 | 100 |
| *Parasenecio* | 2 | 2 | 100 | 100 | 100 |
| *Petasites* | 2 | 2 | 50.0 | 50.0 | 100 |
| *Phoebanthus* | 2 | 2 | 100 | 100 | 100 |
| *Plagiocheilus* | 2 | 2 | 100 | 100 | 100 |
| *Pleurophyllum* | 2 | 2 | 100 | 100 | 100 |
| *Prenanthes* | 2 | 2 | 100 | 0.0 | 0.0 |
| *Pseudobahia* | 2 | 2 | 100 | 100 | 100 |
| *Psilostrophe* | 2 | 2 | 100 | 100 | 100 |
| *Pteronia* | 2 | 2 | 100 | 100 | 100 |
| *Rhagadiolus* | 2 | 2 | 100 | 100 | 100 |
| *Roldana* | 2 | 2 | 100 | 100 | 100 |
| *Synotis* | 2 | 2 | 100 | 100 | 100 |
| *Tephroseris* | 2 | 2 | 100 | 100 | 100 |
| *Trigonospermum* | 2 | 2 | 100 | 100 | 100 |
| *Urospermum* | 2 | 2 | 100 | 100 | 100 |
| *Xanthisma* | 2 | 2 | 100 | 100 | 100 |
