# Supplementary material for: Evaluating the feasibility of using candidate DNA barcodes in discriminating species of the large Asteraceae family
Source: BMC Evol Biol. 2010 Oct 26;10:324. doi: 10.1186/1471-2148-10-324 (PMC3087544; doi:10.1186/1471-2148-10-324)
Supplement: Additional file 3 — Samples in dataset 1 for testing the potential barcodes and accession numbers in GenBank. For each samples in dataset 1, the latin name and accession numbers in GenBank are shown. [file 1471-2148-10-324-S3.DOC]

### Additional file 3 –Samples in dataset1 for testing the potential barcodes and accession numbers in GenBank

| Species | Vourcher Number | *rbcL* | *psbA-trnH* | ITS | ITS2 | *matK* |
| --- | --- | --- | --- | --- | --- | --- |
| *Artemisia anomala* | PS0587MT04 |  |  | GU724268 | GU724310 |  |
| *Artemisia argyi* | PS0590MT01 |  |  | GU724269 | GU724311 | HM989725 |
| *Artemisia argyi* | PS0590MT04 | GQ436428 | GQ435067 | GU724270 | GQ434469 | HM989726 |
| *Artemisia argyi* | PS0590MT05 | GQ436429 |  |  | GQ434470 |  |
| *Artemisia lactiflora* | PS0592MT01 | GU724217 |  |  |  | HM989727 |
| *Artemisia lactiflora* | PS0592MT02 | GU724218 | GU724245 |  |  | HM989728 |
| *Artemisia lactiflora* | PS0592MT03 | GU724219 | GU724246 |  |  | HM989729 |
| *Bidens pilosa var. radiata* | PS0593MT01 | GQ436430 | GQ435068 | FJ980316 | GQ434471 | HM989730 |
| *Bidens pilosa var. radiata* | PS0593MT02 | GQ436431 | GQ435069 | GU724271 | GU724315 | HM989731 |
| *Bidens pilosa var. radiata* | PS0593MT04 | GU724220 |  | GU724272 | GU724316 | HM989732 |
| *Artemisia gmelinii* | PS0594MT01 | GQ436432 | GQ435070 | FJ980317 | GQ434472 |  |
| *Atractylodes lancea* | PS0596MT01 | GU724221 | GU724247 |  |  | HM989733 |
| *Atractylodes lancea* | PS0596MT03 | GQ436433 | GQ435071 |  |  |  |
| *Taraxacum platypecidum var. platypecidum* | PS0598MT01 | GQ436434 | GQ435072 | FJ980318 | GQ434473 | HM989734 |
| *Coreopes tinctoria* | PS0603MT01 | GU724222 |  | GU724273 | GU724319 | HM989735 |
| *Xanthium sibiricum* | PS0604MT01 | GQ436435 | GQ435073 | FJ980319 | GQ434474 | HM989736 |
| *Xanthium sibiricum* | PS0604MT02 |  | GQ435074 | FJ980320 | GQ434475 | HM989737 |
| *Xanthium sibiricum* | PS0604MT03 | GU724223 | GU724248 | GU724274 | GU724337 | HM989738 |
| *Xanthium sibiricum* | PS0604MT04 | GQ436436 |  |  | GQ434476 | HM989739 |
| *Gerbera anandria* | PS0610MT01 | GQ436437 | GQ435075 | GU724275 | GU724326 | HM989740 |
| *Cirsium setosum* | PS0611MT01 | GQ436438 | GQ435076 | GU724276 | GU724318 | HM989741 |
| *Cirsium setosum* | PS0611MT02 | GQ436439 | GQ435077 | GU724277 |  | HM989742 |
| *Cirsium setosum* | PS0611MT04 | GQ436440 | GQ435078 |  | GQ434477 |  |
| *Cirsium japonicum* | PS0612MT02 | GQ436441 | GQ435079 |  | GQ434478 |  |
| *Cirsium japonicum* | PS0612MT03 | GQ436442 | GU724249 | GU724278 | GQ434479 | HM989743 |
| *Cirsium japonicum* | PS0612MT06 |  | GQ435080 |  | GQ434480 |  |
| *Cirsium japonicum* | PS0612MT07 | GQ436443 | GQ435081 |  |  | HM989744 |
| *Farfugium japonicum* | PS0614MT01 |  | GU724250 | GU724279 | GU724325 | HM989745 |
| *Prenanthes macrophylla* | PS0615MT01 | GQ436444 | GQ435082 | FJ980321 | GQ434481 |  |
| *Elephantopus scaber* | PS0617MT01 | GQ436445 | GQ435083 | FJ980322 | GQ434482 |  |
| *Siegesbeckia orientalis* | PS0618MT04 | GQ436446 | GQ435084 | FJ980323 | GQ434486 | HM989746 |
| *Siegesbeckia orientalis* | PS0618MT05 | GQ436447 | GQ435085 | FJ980324 | GQ434487 | HM989747 |
| *Centipeda minima* | PS0620MT01 | GQ436448 | GQ435086 | FJ980325 | GQ434488 | HM989748 |
| *Centipeda minima* | PS0620MT02 | GU724224 | GU724251 |  |  | HM989749 |
| *Centipeda minima* | PS0620MT03 | GQ436449 | GQ435087 | FJ980326 | GQ434489 | HM989750 |
| *Centipeda minima* | PS0620MT04 |  |  |  | GQ434490 |  |
| *Gynura bicolor* | PS0628MT01 | GQ436450 | GQ435088 |  | GQ434491 |  |
| *Carthamus tinctorius* | PS0629MT02 | GQ436451 | GQ435089 | GU724280 | GU724317 | HM989751 |
| *Youngia japonica* | PS0632MT01 | GU724225 | GU724252 | GU724281 | GU724338 | HM989752 |
| *Artemisia annua* | PS0633MT04 |  | GQ435090 | FJ980327 | GQ434492 |  |
| *Artemisia annua* | PS0633MT05 |  |  | GU724282 | GQ434493 | HM989753 |
| *Artemisia annua* | PS0633MT08 |  | GQ435091 | FJ980328 | GQ434494 | HM989754 |
| *Leontopodium leontopodioides* | PS0634MT01 | GQ436452 | GQ435092 | FJ980329 | GQ434495 |  |
| *Ageratum conyzoides* | PS0636MT01 | GQ436453 | GQ435093 | FJ980330 | GQ434496 | HM989755 |
| *Spilanthes paniculata* | PS0640MT01 | GU724226 | GU724253 |  |  | HM989756 |
| *Synedrella nodiflora* | PS0641MT01 | GU724227 | GU724254 | GU724283 | GU724336 | HM989757 |
| *Dendranthema morifolium* | PS0643MT01 |  | GQ435094 | FJ980331 | GQ434499 | HM989758 |
| *Petasites japonicus* | PS0648MT01 |  | GQ435095 | FJ980332 | GQ434501 | HM989759 |
| *Petasites japonicus* | PS0648MT02 | GQ436454 | GQ435096 |  | GQ434502 |  |
| *Galinsoga parviflora* | PS0649MT01 |  | GQ435097 |  | GQ434503 | HM989760 |
| *Eclipta prostrata* | PS0651MT02 |  |  | GU724284 | GU724320 | HM989761 |
| *Eclipta prostrata* | PS0651MT04 | GQ436455 | GQ435098 | FJ980333 | GQ434504 | HM989762 |
| *Eclipta prostrata* | PS0651MT05 | GQ436456 | GQ435099 |  |  | HM989763 |
| *Eclipta prostrata* | PS0651MT06 |  | GQ435100 | FJ980334 | GQ434505 |  |
| *Alternanthera sessilis* | PS0652MT01 | GU724228 |  | GU724285 | GU724307 | HM989764 |
| *Laggera alata* | PS0653MT01 | GQ436457 | GQ435101 | FJ980335 | GQ434506 |  |
| *Ligularia hodgsonii* | PS0655MT01 | GQ436458 | GQ435102 | FJ980336 | GQ434507 | HM989765 |
| *Soliva anthemifolia* | PS0656MT01 | GU724229 | GU724255 | GU724286 | GU724335 | HM989766 |
| *Kalimeris indica* | PS0658MT02 | GU724230 |  | GU724287 | GU724331 |  |
| *Kalimeris indica* | PS0658MT03 | GU724231 | GU724256 | GU724288 | GU724332 |  |
| *Myripnois dioica* | PS0659MT01 | GU724232 | GU724257 |  |  | HM989767 |
| *Artemisia japonica* | PS0664MT02 |  |  | GU724289 | GU724312 | HM989768 |
| *Arctium lappa* | PS0668MT02 | GQ436459 | GQ435105 | GU724290 | GU724308 | HM989769 |
| *Arctium lappa* | PS0668MT05 | GU724233 | GU724258 | GU724291 | GU724309 | HM989770 |
| *Arctium lappa* | PS0668MT09 | GQ436460 |  |  | GQ434509 |  |
| *Inula britanica* | PS0670MT01 | GQ436461 | GQ435106 | GU724292 | GQ434511 | HM989771 |
| *Inula britanica* | PS0670MT02 | GQ436462 | GQ435107 |  | GU724327 | HM989772 |
| *Inula britanica* | PS0670MT03 | GU724234 | GU724259 | GU724294 | GU724328 | HM989773 |
| *Prenanthes tatarinowii* | PS0671MT01 | GQ436463 | GQ435108 | FJ980337 | GQ434512 |  |
| *Eupatorium fortunei* | PS0672MT01 | GQ436464 | GQ435109 | FJ980338 | GQ434513 | HM989774 |
| *Eupatorium fortunei* | PS0672MT02 | GU724235 | GQ435110 | GU724295 | GU724322 | HM989775 |
| *Eupatorium fortunei* | PS0672MT03 | GU724236 | GU724260 | GU724296 | GU724323 | HM989776 |
| *Eupatorium fortunei* | PS0672MT05 | GQ436465 | GQ435111 | GU724297 | GU724324 | HM989777 |
| *Gynura procumbens* | PS0673MT01 | GQ436466 | GQ435112 | FJ980339 | GQ434514 | HM989778 |
| *Rhaponticum uniflorum* | PS0674MT01 | GQ436467 | GQ435113 | FJ980340 | GQ434515 |  |
| *Senecio scandens* | PS0676MT02 | GQ436468 | GQ435114 | FJ980341 | GQ434517 | HM989779 |
| *Senecio scandens* | PS0676MT03 | GQ436469 | GQ435115 | FJ980342 | GQ434518 | HM989780 |
| *Senecio scandens* | PS0676MT05 | GQ436470 | GQ435116 | FJ980343 | GQ434519 |  |
| *Senecio scandens* | PS0676MT06 | GQ436471 | GQ435117 | FJ980344 | GQ434520 |  |
| *Senecio scandens* | PS0676MT07 | GQ436472 | GQ435118 |  |  | HM989781 |
| *Cosmos bipinnata* | PS0678MT01 | GQ436473 | GQ435119 | FJ980345 | GQ434521 | HM989782 |
| *Cosmos bipinnata* | PS0678MT02 | GQ436474 | GQ435120 | GU724298 |  | HM989783 |
| *Aster ageratoides* | PS0680MT01 | GQ436475 | GQ435121 |  | GQ434522 |  |
| *Gnaphalium affine* | PS0684MT01 | GQ436476 | GQ435122 | FJ980346 | GQ434523 | HM989784 |
| *Tragopogon porrifolius* | PS0686MT01 | GQ436477 | GQ435123 | FJ980347 | GQ434524 | HM989785 |
| *Gaillardia pulchella* | PS0687MT01 | GQ436478 | GQ435124 | FJ980348 | GQ434525 | HM989786 |
| *Gaillardia pulchella* | PS0687MT02 | GQ436479 | GQ435125 | FJ980349 | GQ434526 | HM989787 |
| *Dichrocephala benthamii* | PS0697MT01 | GQ436480 | GQ435127 | FJ980350 | GQ434529 | HM989788 |
| *Inula japonica* | PS0698MT04 | GU724237 | GU724261 | GU724299 | GU724329 | HM989789 |
| *Inula japonica* | PS0698MT05 | GU724238 | GU724262 | GU724300 | GU724330 | HM989790 |
| *Saussurea involucrata* | PS0699MT01 | GQ436481 | GQ435128 | FJ980351 | GQ434530 |  |
| *Scorzonera austriaca* | PS0700MT01 | GQ436482 |  | GU724301 | GQ434531 |  |
| *Inula cappa* | PS0701MT01 |  | GQ435129 | FJ980352 | GQ434532 |  |
| *Inula cappa* | PS0701MT02 | GQ436483 | GQ435130 |  |  |  |
| *Artemisia lavandulaefolia* | PS0703MT01 | GQ436484 | GQ435131 | FJ980353 | GQ434533 |  |
| *Dendranthema lavandulifolium* | PS0704MT01 | GQ436485 | GQ435132 | FJ980354 | GQ434534 | HM989791 |
| *Vernonia cinerea* | PS0708MT01 | GU724239 | GU724263 |  |  |  |
| *Emilia sonchifolia* | PS0709MT01 | GQ436486 | GQ435134 |  | GQ434535 | HM989792 |
| *Emilia sonchifolia* | PS0709MT02 | GQ436487 | GQ435135 | FJ980355 | GQ434536 | HM989793 |
| *Emilia sonchifolia* | PS0709MT03 | GQ436488 | GQ435136 | FJ980356 | GQ434537 | HM989794 |
| *Emilia sonchifolia* | PS0709MT04 | GU724240 | GU724264 |  |  | HM989795 |
| *Erigeron annuus* | PS0710MT01 | GU724241 | GU724265 | GU724302 | GU724321 | HM989796 |
| *Artemisia scoparia* | PS0711MT01 | GU724242 |  | GU724303 | GU724313 | HM989797 |
| *Saussurea nivea* | PS0713MT01 | GQ436489 | GQ435137 | GU724304 | GU724334 | HM989798 |
| *Parthenium hysterophorus* | PS0714MT01 | GU724243 |  | GU724305 | GU724333 | HM989799 |
| *Achillea wilsoniana* | PS0715MT01 | GQ436490 | GQ435138 | FJ980357 | GQ434539 | HM989800 |
| *Achillea wilsoniana* | PS0715MT02 | GQ436491 | GQ435139 | FJ980358 | GQ434540 | HM989801 |
| *Achillea wilsoniana* | PS0715MT03 | GU724244 | GU724266 |  |  | HM989802 |
| *Aster tataricus* | PS0721MT01 |  | GU724267 | GU724306 | GU724314 | HM989803 |
| *Aster tataricus* | PS0721MT04 | GQ436492 | GQ435142 | FJ980359 | GQ434543 | HM989804 |
