# Supplementary material for: Evaluating the feasibility of using candidate DNA barcodes in discriminating species of the large Asteraceae family
Source: BMC Evol Biol. 2010 Oct 26;10:324. doi: 10.1186/1471-2148-10-324 (PMC3087544; doi:10.1186/1471-2148-10-324)
Supplement: Additional file 4 — Accession numbers of the five loci sequences from GenBank for the meta-analysis. For each samples used for the meta-analysis, the accession numbers in GenBank are shown. [file 1471-2148-10-324-S4.DOC]

**Additional file 4 –Accession numbers of the five loci sequences from GenBank for the meta-analysis**

EU677055,AB436862,AF459990,AB435100,EF538325,AY914815,EU677054,AB436859,AF460026,AB435116,EF538324,EF210929,EU677044,AB436854,AF460028,AB196600,EF538323,EF210970,EU841130,AB436893,AF459993,AB196598,EF538322,EF210931,EU841127,AB436888,EF537915,AB196605,EF538321,EF210965,EU841117,EF537950,FJ697079,AB196611,EF538320,EF210949,EU841104,EF537949,FJ697085,AB196612,EF538317,EF210945,EU841103,EF537947,FJ697084,AB196613,EF538316,EF210943,EU841113,EF537945,FJ697165,AB196607,EF538311,EF210958,EU385004,EF537944,FJ697164,AB196608,EF538310,DQ444719,EU385007,EF537938,FJ697163,AB196599,EF538308,EF556360,EU384985,EF537935,FJ697162,AB196597,EF538307,EF556359,EU384972,EF537934,FJ697081,AB196609,EF538306,DQ444731,EU384955,EF537927,FJ697080,AB196610,EF538305,DQ444730,EU384956,EF537926,FJ697078,AB196596,EF538302,DQ444717,AM234875,EF537925,AY297645,AY554114,EF538301,EF420925,AB530974,EF537924,FJ697077,AY554111,EF538299,EF420945,FJ395566,EF537923,FJ697109,AY554110,EF538298,AY914822,DQ006065,EF537921,FJ697108,AY554109,EF538296,EF420938,EU385011,EF537919,AF391694,AY554108,EF538295,EF420933,EU841124,EF537941,AF391693,AY554107,EF538399,EF420916,EU841119,EF537917,AF391692,AY554106,EF538292,EF420917,EU841120,EF537916,AF391699,AY554104,EF538291,EF420944,EU841116,EF537914,AF391698,AY554103,EF538288,EF420924,EU841121,EF537913,AF391724,AY554102,EF538286,EF420931,EU841115,EF537912,AF391723,AY554101,EF538284,EF420915,EU841118,EF537911,AF391722,AY554100,EF538283,EF420935,EU384960,EF537910,AF391727,AY554099,EF538282,AB118120,EU841108,EF537920,AF391726,AY554098,EF538281,EF420926,EU841109,EF537933,AF391725,AY554096,EF538280,EF420942,EU841106,EF537943,AF391733,AY554095,EF538278,EF420927,EU841110,AB262026,AF391732,AY554094,EF538277,EF420930,EU841102,AB262025,AF391731,AY554093,EF538274,AF528475,EU841098,AB262024,AF391697,AY554092,EF538273,AF528474,EU384948,AB262046,AF391696,DQ451821,EF538272,AF528473,EU385010,AB262045,AF391695,AM117053,EF538267,AF528472,EU385002,AB262044,FJ697133,AM117051,EF538263,AF528471,AB530957,AB262043,FJ697132,DQ451786,EF538261,AF528470,EU384973,AB262042,FJ697131,DQ451783,EF538260,AF528469,EU676918,AB262041,AY554090,DQ451817,EF538259,AF528452,EU676916,AB262040,AY554088,AY554105,EF538255,EF210954,EU676915,AB262039,AY554087,AM117045,EF538253,AF496999,FJ395580,AB262038,AY554073,AM117044,EF538249,DQ383874,AY395545,AB262037,AY554069,AY327537,EF538243,AF497002,FJ395583,AB262036,AY554061,AY327531,EF538242,DQ383871,AY395542,AB262035,AF456779,AB118128,EF538241,DQ383861,AB530976,AB262034,DQ451730,AY914825,EF538240,AF497003,DQ355129,AB262033,AJ633230,AB118111,EF538239,AF465853,EU841105,AB262027,AY554075,AB118139,EF538236,DQ383856,EU677048,DQ023004,AY554079,AB118132,EF538235,EF210963,GU135190,DQ451736,AY554071,EF420920,EF538229,AF229309,AB530960,DQ451739,AY554052,AY879183,EF538228,AF229300,FJ395571,DQ451732,EU049355,AY155613,EF538225,AF229306,AY395562,DQ451722,EU049352,AY155612,EF538220,AF229308,EU385018,DQ451724,EU049353,AY155611,EF538219,AM690710,EU677032,DQ451723,EU049354,AY155608,EF538218,AF229302,AB530972,DQ451701,EU841331,AY327538,EF538215,AF229307,AY395563,DQ451700,EU841348,AY155605,EF538214,AF229303,EU841122,DQ451697,EU385342,AY155604,EF538213,DQ028911,EU384969,DQ451720,EU385327,AY155603,EF538212,AY012320,AY874430,DQ451696,EU841319,AF528495,EF538210,AY012267,AB530959,DQ451692,AF391709,AF528490,EU667473,AY012266,AY874429,DQ451731,AF391708,AF528487,EF538209,AY012265,EU384954,DQ451727,AF391707,DQ451767,EU667502,AY012262,AB530953,DQ451698,AF391715,DQ451766,EU667500,FJ459682,EU385027,DQ451713,AF391714,AF528484,EF538207,AY012261,L13647,DQ451702,AF391713,AF528486,EU667474,AY012260,L13076,DQ451710,AF391712,FJ980351,EF538204,AY012258,L13863,DQ451709,AF391711,AB196595,EF538203,AY012256,AB530980,DQ451699,AF391710,AB196602,EF538200,AY012254,L13645,DQ451695,AF391718,AB435122,EF538198,AY012253,EU384938,DQ846565,AF391717,AM774450,EF538197,AY012252,AF097517,DQ846564,AF391716,DQ915861,EF538196,FJ459667,AB530968,AB217689,AF391721,AY953933,EF538192,AY012249,EU384953,EF537937,AF391720,AF459922,EF538190,AY012246,AB530978,AY929880,AF391719,AF459923,EF538189,AY012241,DQ006056,AY929879,AF391753,AF459929,EF538186,AY012240,DQ006055,AY929878,AF391752,AF459930,EF538182,AY012239,EU749446,AY929877,AF391751,AF459931,EF538180,AY012237,EU749445,AY929876,AF391750,AF459933,EF538178,AB118124,EU749435,AY929875,AF391749,AF459934,EF538176,AF319116,EU841357,AY929874,AF391748,AF459935,EF538174,AF319114,EU841354,AY929873,AF391747,AF459937,EF538170,AB118127,EU841339,AY929872,AF391746,AF459938,EF538168,AF257787,EU841325,AY929871,AF391745,AY155609,EF538166,AF257788,EU841324,AY929870,AF391744,AF459946,EF538164,AF228406,EU841335,AY929869,AF391740,AF459947,EF538161,AF228405,EU385382,AY929868,AF391739,AF459948,EF538160,AF228404,EU385385,AY929867,AF391738,AF459951,EF538159,AF228403,EU385364,AY929866,AF391737,AF459953,EF538151,AF228401,EU385351,AY929865,AF391743,AF459927,EF538149,AF228399,EU385333,AY929864,AF391742,AF459954,EF538144,AF228398,EU385334,AY929863,AF391741,AF459955,AM774472,AF228397,EU385360,AY929862,AF391756,AF459956,AM774471,AF228395,AF460012,AY929861,AF391755,AF459957,AM774469,AF228390,AF460009,AY929859,AF391754,AF459958,AM774467,AF228389,FJ697076,AY929858,AF391730,AF459970,AM774464,AF156016,EU385389,AB436881,AF391729,AF459960,AM774463,AY914827,EU841349,AB436879,AF391728,AF459961,AM774461,AF118537,EU841342,AB436876,AF391703,AF459962,AM774458,EU007683,EU841343,AB436865,AF391702,AF459963,AM774452,EU007681,EU841338,AB436861,AF391701,AF459965,AM774447,EU007680,EU841344,AB436857,AF391700,AF459966,AM774445,EU007679,EU841337,AB436894,AY551501,AF459969,EF538227,EU007678,EU841341,AB436850,EU049365,AF459972,EF538226,EU007677,EU385338,AB436846,AF318911,EF538175,EF538172,EU007676,EU841328,AB436863,AF391736,AF465894,EF538372,EU007675,EU841332,AB196630,AF391735,FJ696980,EF538354,AF061906,EU841327,AB196629,AF391734,AF465873,EF538338,AF061913,EU841333,AB196628,AF391691,FJ697060,EF538297,AF061895,EU841323,AB196626,AF391690,AF465872,EF538254,AF061908,EU841316,AB196625,AY785125,FJ697072,EF538223,AF061910,AY013522,AB196624,AY013516,AF465871,EF538163,AF061894,EU385388,AB196623,AY785113,FJ696977,EF107658,AF061911,EU385380,AB196622,AY013501,AF465869,EF107657,AF061883,FJ395389,AB196621,AY013498,AF465867,EF108398,AF061884,EU385352,AB196620,AY013492,AF465847,EF108396,AF049285,EU749311,AB196619,AY013491,AF465874,EF108392,AF049281,EU749309,AB196617,AY013490,FJ697004,EF091598,AF049279,EU749308,AB196631,AY013542,FJ697003,EF091590,EF210968,AF459995,AY554086,AY013556,AF391561,EF091589,AF049254,AJ633218,AY554083,AY013552,AF391560,EF091582,U95284,FJ395390,AY554082,AY013513,AF391559,DQ915898,U95285,AJ633231,AY554081,AB118154,AF391566,DQ915866,EU007672,AF151515,AY554080,AY785097,AF391565,AM398916,U95286,AB457364,AY554077,AY013537,AF391591,DQ862119,EU007682,AF456782,AY554076,AY013535,AF391590,DQ862118,U95280,AF151441,AY554068,AY013534,AF391589,AB259353,U95294,GU135027,AY554067,AY013502,AF391594,AB259352,U95283,EU049361,AY554066,AY013532,AF391593,AB259351,U95278,FJ395377,AY554065,AY013529,AF391592,AB259350,EF210971,AJ633157,AY554064,AY013527,AF391600,AB259349,DQ444718,AJ633279,AY554063,AY013526,AF391599,EU667523,EF210966,DQ022963,AY554062,AY013525,AF391598,EU667515,EF210964,FJ640024,AY554059,AY013523,AF391564,DQ414734,AF049256,FJ395443,AY554058,AY013521,AF391563,AB284129,AF229301,EU841347,AY554057,AY013518,AF391562,DQ198259,AM493998,EU385348,AY554056,AY009467,FJ697045,EF091576,DQ889642,AF456785,AY554055,DQ923845,FJ697044,EF091584,DQ444736,AY551490,AJ633226,DQ923851,AF465870,AY914813,DQ383885,EU841334,AJ633268,DQ923850,AF422139,AY876283,AF496998,EU385332,AJ633271,AY785092,AF422135,AY876282,L32922,AF151446,DQ451716,DQ923849,AF422134,AY876281,FJ980347,EU385407,AJ633221,AJ633228,AF422121,AY876280,AM493996,AJ633257,AJ633153,AJ633224,AF422112,AY876279,AM493995,AF456802,AY554074,DQ451725,AF422111,AY876278,AM690738,AY554072,AJ633265,AJ633223,AF422110,AY876277,AM690715,AJ633131,AJ633272,DQ451750,AF528491,AY876276,AY914812,AF460029,AY327535,AJ633215,AF422123,EF107651,FJ861546,EU385315,AY327529,AJ633164,AF422122,EF108393,FJ861545,AJ429380,AB118151,DQ923852,AF422132,FJ980346,FJ861543,AF151469,AB118143,DQ923848,AF422118,EF108394,FJ861542,EU385331,AB118142,AY013494,AF422109,AY953936,FJ861539,AY013520,AB118153,AY013512,AF330095,AY953905,FJ861533,GQ248078,AB118152,AF459998,AF330088,EF538265,FJ861532,GQ248077,AB118147,DQ208166,AF330102,EF538264,FJ861531,EU781139,AJ633170,DQ208170,AF330101,AJ831538,FJ861530,EU781138,AY156990,DQ208172,AF412871,EF577285,FJ861515,EU781418,AY156989,DQ846529,AF412839,EF577284,FJ861521,EU841171,AY156988,DQ846528,AF412862,AY548202,FJ861517,EU841169,AY156991,AB436891,AF412884,AY548201,FJ861518,EU841158,AY156982,AB436849,AF412887,AB212735,FJ861516,EU841146,AY156986,EF537951,AF391576,DQ925700,FJ861519,EU841145,AY156981,EF537918,AF391575,DQ028903,FJ861520,EU841154,AY156980,AJ633250,AF391574,EF538244,FJ861522,FJ861477,AF542066,AY013493,AF391582,AY275656,FJ861525,EF530239,AJ633229,AY785093,AF391581,AY275655,FJ861555,EF155776,DQ451728,DQ923843,AF391580,EF091587,FJ861551,EF155770,DQ451705,AF318910,AF391579,AM398882,FJ861549,EF155753,DQ451704,AF456799,AF391578,DQ028914,FJ861548,DQ355904,DQ451703,AF459984,AF391577,AY169242,FJ861524,EU527231,AJ633220,AF459985,AF391585,EF107650,FJ861541,AF459950,AB118148,AF459986,AF391584,AF539955,FJ861535,AF459944,AB196632,AF459988,AF391583,AF539956,FJ861547,FJ696962,AB196616,AF460025,AF391588,AF539957,FJ861526,AF429846,AB436867,AB032896,AF391587,AF539954,FJ861556,AF412875,AF456787,EU049344,AF391586,AF539953,GU216578,AF412846,AF460005,AF456775,AF391619,AF539933,GU216577,EU841161,AF460027,AY554084,AF391618,AF539930,GU216568,AF412854,AF459973,AY156987,AF391617,AF539928,GU216567,AF412844,AF459994,AF460007,AF391616,AF539941,GU216579,AF412840,AF459999,AF460010,AF391615,AF539942,GU216565,AF412845,AF460000,EF537930,AF391614,AF539925,GU216583,AF412864,AF460001,AF460021,AF391613,AF539926,GU216582,AF412869,AF460002,DQ208165,AF391612,AF539929,GU216566,AF412867,AF459975,AY013554,AF391611,AF539927,GU216573,AF412870,AF460003,AY013551,AF391610,AF539923,GU216572,AF412865,AF460004,AY013550,AF391606,AF539952,GU216571,AF412879,AF459977,AY013549,AF391605,AF539948,GU216564,AF412838,AY156985,AY013545,AF391604,AF539947,GU216563,AF319104,AF459979,AY013541,AF391603,AB355526,GU216561,EF530226,AF459980,AJ633159,AF391609,AF539949,GU216560,AM774465,AF459981,AF459996,AF391608,AF539946,GU216570,EF091600,AF460013,EF537909,AF391607,AF539940,GU216569,AF118488,AF460015,AY929860,AF391622,AF539939,GU216575,AF118490,AF459974,AJ633242,AF391621,AB355525,GU216574,EF107653,AF460017,AJ633246,AF391620,EF577313,GU216562,AF118489,AF460018,DQ208169,AF391597,EF577272,GU216555,AF422136,AF460019,AY554089,AF391596,AY117466,GU216554,DQ451770,AF459982,FJ395405,AF391595,AF501626,GU216581,AF528458,AF459983,AY013497,AF391570,AF459932,GU216580,AF528457,AF459992,AY013496,AF391569,EF107652,FJ845877,DQ862121,AF460022,AY009461,AF391568,EF538194,FJ845876,AB457301,AF460023,AY009459,AF391567,AF459968,FJ845875,AF412866,AF460024,AY009458,AY017366,AB245093,FJ845874,EU200229,AF459989,AY009462,AY017356,EF108402,FJ845873,U74441,AM690600,AY009466,FJ457937,AF465890,GU216576,FJ980345,AM690570,AY009460,AF391602,FJ697065,FJ459718,L48338,AM690589,AF456781,AF391601,FJ697064,FJ459715,AY548211,AM690586,AB196618,AF229290,FJ697063,FJ459708,L48304,AM690563,AB008761,AF229288,FJ697062,FJ459707,L48302,AM690595,AB008758,AF229292,FJ697061,FJ459699,FJ639910,AM690580,AB436853,AY012340,FJ696978,FJ459698,AM493997,AB234745,DQ451706,AY012339,EF108404,FJ459697,EF530256,AB234744,AF460006,AY012330,EF108403,EU846423,AF412874,AB234743,AF460008,AY012318,AY169241,EU750596,AF412873,EF091618,AF151481,AY012314,FJ697005,EU750595,U74442,EF091615,AF151473,AY012312,FJ696965,EU750585,AF412868,EF091614,EU841326,AY012309,FJ696964,EU841307,DQ319132,AB234761,AF456776,AY012307,AF429878,EU841304,AF422114,AB234760,FJ697074,AF319132,AF422117,EU841306,AF319133,EF091607,AY009465,AF319152,AF422115,EU841290,AM493994,EF091605,AJ633247,AF319150,AF422128,EU841276,AF092611,FJ459713,DQ023036,AY012334,AF422131,EU841275,AF422120,FJ459701,DQ023011,AF319147,AF422133,EU841282,AF118917,FJ459726,DQ023035,AF319125,AF422130,FJ861523,AF459971,FJ459725,DQ023034,AF319123,AF422129,EF556404,EU796891,FJ459724,DQ023013,AF319120,AF422124,EF556403,AF047927,FJ459723,DQ023033,AF319119,AF422119,EF556402,AF047924,FJ459722,DQ022981,AF319118,AF422113,EF556401,AF140459,FJ459721,DQ022980,AF319115,AF422125,EF556400,AF319102,FJ459720,DQ022989,AF319112,DQ862120,EF556393,DQ005970,FJ459719,DQ022967,AF319110,AF386493,EF556392,DQ005969,FJ459717,DQ507984,AF319109,EF091575,EF556390,EU676927,FJ459716,DQ022998,AF319108,AJ312823,EF556389,EU676926,FJ459714,DQ022971,AF319105,AJ831534,EF556388,EU677055,FJ459712,DQ022984,AF319103,AF314594,EF556387,EU677053,FJ459711,DQ023026,AF319100,AF314602,EF556386,EU677052,FJ459710,DQ023032,AF315080,AF314591,EF556385,EU677047,FJ459706,DQ507995,AF140483,AF314604,EF556384,EU677046,FJ459705,DQ507986,AF140479,AF314600,EF556383,EU677045,FJ459704,DQ840450,AY826247,AF314601,EF556382,EU677043,FJ459702,DQ023017,AF140475,AF314592,EF556380,EU677042,FJ459700,DQ023016,AF140461,AF314596,EF556377,EU677039,AY914848,DQ023008,DQ451820,AJ831535,EF556376,EU677038,EU571432,DQ023023,DQ451823,AF319126,EF556379,EU677037,EU571446,DQ023001,DQ451794,AF422127,EF556378,EU841128,EU571447,DQ022974,AF528489,AF229271,EF556375,EU677031,EU571449,DQ023015,DQ451824,AM774473,EF556374,EU677030,FJ395501,DQ023030,AF528494,EF577323,EF556370,EU677029,EF420908,DQ022997,AY879153,EF577322,EF556369,EU385025,EF420882,DQ022976,AF140457,AJ864607,EF556368,EU385021,EF420883,DQ840451,AF140455,EU179212,EF556367,EU385019,EU547790,DQ508001,AF140453,EU179214,EF556366,EU384994,EU547792,DQ023029,AF140449,FJ969855,EU007660,EU385017,DQ917443,DQ023028,AF459928,AF247098,EU007665,EU384941,DQ917449,AF151460,AJ400785,AF218884,EF420913,EU385014,DQ917448,AB436855,AJ400781,EU667521,EF420886,EU385013,DQ917447,AB436895,AJ400779,AF156020,EF420910,EU385006,DQ917450,AB436886,DQ319103,AF386498,EF420905,EU385003,DQ917446,AB436851,AM114322,DQ028913,EF420904,EU384998,DQ006148,AB436848,AB435147,AM774453,EF420902,EU384997,DQ846181,AB436874,AB435103,AM774451,EF420911,EU384995,DQ846180,AB436852,AJ400093,EF577293,EF420885,EU384993,FM998705,AB436843,AJ400078,EF577287,EF420912,EU384992,EU531700,AB436873,AF386494,EF091579,EF420894,EU384991,EU531702,AJ633258,AF140435,EF577273,FJ459709,EU384990,FM998696,DQ507979,AF140433,EF114672,EF210982,EU384989,FM998704,DQ840441,AF140429,AY548210,EF211001,EU384988,FM998688,DQ840440,EU667512,AF061391,EF211003,EU384980,FM998683,DQ022994,FJ457927,AF061390,EF210977,EU384979,FM998682,DQ022993,AF156024,EU621367,EF210979,EU384976,EF211000,DQ840442,AF156013,AF422126,EF210981,EU384975,EF210993,DQ840443,AF156002,AF386503,EF211009,AB530965,EF210991,AJ633241,AF155999,AF386502,EF211008,EU384967,EF538088,DQ508012,AF155998,EF108397,EF211005,EU384965,DQ917445,DQ508011,DQ629024,AF079946,EF210996,EU384951,DQ917441,DQ022964,AF165840,AM269944,EF210995,EU384950,EF538069,AJ633280,AJ296417,AM269943,EF210990,EU384947,EF538060,AY551477,AY554112,AM269942,EF211004,EU384946,EF538031,EU049345,AY155610,AM269941,EF211007,EU384942,AB234783,AY785099,AF459941,EF107649,EF211010,EU384943,FJ395487,AY013503,AF459945,EF538251,EF210988,EU384940,EF091629,DQ846630,EF538276,AM269945,EF210987,EU385024,EF091603,AY013495,AF459959,AM398847,EF210985,EU385015,EU547793,AY013533,AJ400806,AF386497,EF210984,EU384963,AB234775,AY013489,AF319151,EF538158,EF210983,EU384958,FJ395449,AF460020,AF319148,AJ831533,EF211020,EU213449,AY155657,EU385400,AF319143,AJ831532,EF210998,EU213448,DQ131866,AY009464,AF319142,AJ831531,EF210989,EU213447,EF538040,FJ789805,AF319139,AJ831530,AM690559,EU384999,AY155641,FJ697046,AF319131,AJ831529,AM690556,EU841132,AF129852,FJ697028,EU637252,AJ831528,AM690569,EU677041,AY914849,FJ697027,AF459925,AB359077,AM690583,EU677040,AF129851,FJ696998,EF538143,EF577308,AM690560,EU677028,AF129850,FJ696997,AY929883,EF577305,AM690555,EU677027,AF129849,FJ697029,AF386495,EF577303,AM690565,EU677026,AF129848,FJ697049,AF386496,EF577296,AM690596,EU677021,AF129847,FJ697048,EF538396,EF538318,AM690579,EU677020,AF129845,FJ696986,AF459924,EU667492,AM690576,EU384968,EU750459,FJ697023,DQ319138,EF577290,AM690564,EU042186,DQ131883,FJ697022,AF058874,EF577282,AM690561,EU385008,EU337692,FJ697059,AF058869,EF577299,AM690557,EU384945,EU337691,FJ697058,AF047971,GU188570,AM690582,EU384944,EU007671,FJ696990,AF047972,EF108400,AM690567,AM234893,EU007670,FJ696988,AF047968,EF538326,AM690581,AM234858,EU007669,FJ697038,AF047970,EU667477,AM690575,EU385028,EU007662,FJ697037,AF047962,EF538169,AM690574,EU677024,EU007661,FJ697034,AF047900,GQ396673,AM690573,DQ006066,EU007659,FJ697033,DQ479106,FJ528302,AM690578,AM234862,EU007658,FJ697054,AB196601,EF577300,AM690577,EU384983,AY611224,FJ697053,DQ159944,AY548200,AM690584,EU385016,AY155666,FJ696994,AY548205,EF577316,AM690558,AB530961,EF538021,FJ696993,AB435106,DQ826453,AM690566,EU385009,EU341886,FJ696992,DQ451771,FJ696963,AM690594,AM234879,EU341887,FJ697069,AF459940,EF114670,AM690572,EU384984,EU341885,FJ697071,AF459943,FJ696981,AM690592,EU841101,EU341884,FJ697070,AJ296447,FJ697066,AM690590,AM234881,EU341882,FJ697019,EU239682,FJ697073,AM690571,EU384982,EU341881,FJ697018,EU841147,FJ696979,EF538057,AB530970,EU341880,FJ696973,AF061376,FJ696961,EF538050,EU385012,EU341878,FJ696972,U74425,EF108399,EF538074,EU385005,EU341877,FJ696985,AF047957,EF577277,EF538066,EU385000,FJ493263,FJ696984,U69706,EU179216,EF538086,EU384978,FJ395482,FJ697068,L48320,EF577309,EF538077,EU384970,EF538071,FJ697067,L48318,EF577306,EF538079,EU384961,FJ395504,FJ697001,L48336,EF577304,EF538065,EU384957,FM998713,FJ697000,L48334,EF577302,EF538084,EU384977,FM998709,FJ696983,L48330,EF577301,EF538063,EU384966,FM998691,FJ696982,L48324,EF577298,EF538081,EU676925,FM998684,FJ697036,L48328,EF577297,EF538083,EU676924,EF211015,FJ697035,L48326,EF577295,EF538067,EU676923,AY611217,FJ696989,L48316,AM269946,EF538059,EU676922,AY611211,FJ696976,L48314,EF108395,EF538054,DQ006064,AY611227,FJ696975,L48296,EF577320,EF538037,EU676921,AY611221,FJ697040,L48172,AF422140,EF538036,EU676920,AY611220,FJ697039,L48132,EU785941,EF538053,EU676919,AY611219,FJ697032,L48312,EF577319,EF538035,EU676917,AY611223,FJ697031,L48124,AJ563924,EF538034,EU385029,AY611218,FJ697052,L48130,EF577317,EF538032,EU676929,AY611234,FJ697051,L48310,EF577292,EF538070,EU676928,FJ395488,FJ697057,L48298,EU179215,EF538064,EU677023,EF211017,FJ697056,AF422137,EF577286,EF538028,EU677022,DQ444762,FJ696971,L48170,AJ831537,EF538027,AM234918,EF211014,FJ696970,L48168,AJ297261,EF538025,EU677051,FM998656,FJ697010,L48128,EF577288,EF538024,EU677050,EF211012,FJ697009,L48122,EF577289,EF538062,EU677049,EU547791,FJ697030,L48126,AY548204,EF538056,GU135265,FM998672,FJ639960,L48120,GU011987,EF538022,EU677036,EU841277,FJ639959,L48116,EF107656,EF538030,EU677035,EU750589,FJ639958,L48174,EU179213,EF091628,EU677034,GU135356,FJ639955,L48292,FJ528301,EF091626,EU677033,DQ006149,FJ639953,L48134,AY548199,EF091617,AM234916,FJ493264,FJ639952,L48306,FJ539127,EF091616,EU385020,FJ395471,FJ639949,L48308,FJ528300,EF091613,AY874431,AY457987,FJ639951,L48290,FJ861510,EF091606,AB530964,AY457989,FJ639944,L48288,FJ861509,EF091604,AY395534,EF374262,FJ639961,U95290,FJ861508,EF538068,EU384949,AM690599,FJ639928,AB435109,FJ861503,EU846438,L13643,FJ395553,FJ639926,AB435120,FJ861502,EU846416,L13652,EF374281,FJ639921,AB435142,FJ861501,EF538073,L13641,EU846396,AB457326,AB435375,FJ861497,DQ444781,L13650,DQ444780,AB457325,AB435102,FJ861496,EU846395,L14073,DQ383919,AB457322,AB435125,FJ861495,DQ444779,AY874437,DQ383918,AB457320,AB435105,FJ861471,EU846394,AB530982,EU841297,AB457318,AB435096,FJ861472,DQ444778,L13929,EU841296,AB457316,AB435124,FJ861473,DQ444777,EU749320,EU531699,AB457313,AM493990,FJ861474,DQ444776,EU749319,AF129843,AB457312,L48164,FJ861479,DQ444760,EU749444,EU841286,AB457311,L48166,FJ861475,DQ444773,EU749443,DQ846282,AB457310,L48152,FJ861476,DQ444772,EU749438,EU571426,AB457308,L48158,FJ861478,DQ444770,EU749437,DQ006144,AB457304,L48156,FJ861493,DQ444769,EU749436,FJ418749,AB457302,L48148,FJ861487,DQ444768,EU749434,DQ006143,AB457296,L48146,FJ861485,DQ444767,EU749433,DQ131867,AB457293,L48144,FJ861483,DQ444766,EU749430,DQ131868,AB457292,L48142,FJ861482,EU846414,EU749429,EF374280,AB457291,L48140,FJ861480,DQ444765,EU749428,EF374279,AB457279,L48138,FJ861506,EF420901,EU841355,EF374278,AB457278,L48136,FJ861494,EF420887,EU749423,EF374277,AB457277,EU117267,FJ861499,FJ031849,EU749422,FJ493262,AB457276,AF165829,FJ861498,DQ383917,EU749421,EF538023,AB457274,AJ831536,FJ861511,DQ383915,EU385405,DQ131853,AB457271,DQ319123,FJ459676,EF133507,EU385401,FJ395492,AB457269,AM087191,FJ459672,DQ383914,EU385398,AF129842,AB457268,AF058858,FJ459660,DQ383913,EU385373,AM690598,AB457267,AF319117,FJ459659,DQ383902,EU385396,AM690568,AB457266,AY012306,FJ459649,DQ383901,EU385318,AY914834,AB457256,L33459,FJ459648,DQ383900,EU385392,AF129838,AB457254,EF155799,FJ459647,DQ383899,EU385391,EF590670,AB457251,AF047953,EU527192,DQ383898,EU385384,DQ006142,AB457249,FJ969854,EU841170,DQ383897,EU385381,GQ248244,AB457248,FJ789806,EF556358,DQ383896,EU385376,EF590669,AB457247,FJ697047,EF556357,DQ383894,EU385375,DQ006141,AB457244,FJ696999,EF556356,DQ383893,EU385374,AY874427,AB457243,FJ697050,EF556355,DQ383892,EU385372,L13862,AB457239,FJ696987,EF556354,DQ383888,EU385371,EF590498,AB457238,FJ697026,EF556347,DQ383908,EU385370,GQ248547,AB457237,FJ697025,EF556346,DQ383905,EU385369,EF590497,AB457236,FJ697024,EF556345,DQ383904,EU385368,EU729341,AB457234,FJ696991,EF556343,DQ383887,EU385367,EU729344,AB457330,FJ697055,EF556342,DQ846217,EU385359,EU729343,AB457329,FJ696996,EF556341,DQ846216,EU385358,EF538309,AB435153,FJ696995,EF556340,EF091602,EU385356,AM774466,AB435152,FJ697021,EF556339,AB234774,EU385355,AM774462,AB435151,FJ697020,EF556338,AB234772,EU385353,EU667517,AB435148,FJ696974,EF556337,AB234771,EU385346,FJ457944,AB435146,FJ697002,EF556336,AM690562,EU385344,EU527195,AB435141,FJ697043,EF556335,DQ006147,EU385329,FJ457929,AB435140,FJ697042,EF556331,AY914852,EU385328,FJ457931,AB435138,FJ697041,EF556330,AY914851,EU385324,EU667480,AB435137,FJ697013,EF556333,AY914850,EU385323,DQ005975,AB435134,FJ697012,EF556332,AY914836,EU385319,DQ005983,AB435131,FJ697011,EF556329,AY914846,EU385320,AJ864594,AB435129,EU979538,EF556328,DQ874336,EU385317,EU527221,AB435126,AB457317,EU527228,AY914839,EU385404,EU527217,AB435123,AB457273,EF556323,AY914838,EU385393,DQ444720,AB435121,AB457259,EF556322,AY914840,EU385341,EU841148,AB435119,AB457257,EF556321,AY914835,EU385336,EF530230,AB435117,AB457250,EF556320,DQ006156,EU214220,AY046937,AB435115,EU667495,EU007674,EF538076,EU214219,AF172020,AB435113,EU667514,EU007673,AY914837,EU214218,AF165832,AB435108,EU667511,EF420951,EF210978,EU385377,EF577315,AB435107,EU667475,EF420922,EF211016,EU841358,EF091578,AB435145,EU667471,EF420947,EF210980,EU749432,AF156009,EF538411,EU667494,EF420940,EF211013,EU749431,AF140484,EF538405,EU667484,EF420939,EF210997,EU749420,AJ404744,EF538386,EU667499,EF420937,EF210994,EU749419,DQ391195,EF538366,EU667526,EF420949,EF210992,EU749418,DQ005977,EF538363,EU667469,EF420921,EF211006,EU749413,AF172019,EF538346,EU667530,EF420950,EU531698,EU749412,U74407,EF538303,EU667468,EF420929,FJ418750,EU385347,EU841164,EF538294,EU667503,FJ459662,DQ444763,EU385330,AF412855,EF538266,EU667486,EF210933,EF556406,EU385386,AM087164,EF538257,EU667466,EF210953,EF556405,EU385322,AF494007,EF538250,EU667490,EF210955,DQ444775,EU385321,AF493996,EF538248,EU667462,EF210928,DQ444774,EU385379,EU200225,EF538238,EU667507,EF210930,DQ444761,EU385337,DQ005972,EF538222,EU667504,EF210932,DQ444764,EU385408,AF079935,EF538193,EU667493,EF210961,EF420889,EU749417,U74448,EF538357,EU667491,EF210960,EF420907,EU749416,AF092631,EF538181,EU667482,EF210957,AY914842,EU385340,FJ457947,EF538179,EU667527,EF210948,AY155647,EU385362,AF412881,EF538167,EU667508,EF210947,AY327536,AJ633254,EF483943,EF538162,EU667496,EF210941,AY327530,AJ633141,U74438,EF538156,EU667531,EF210956,EF420903,EU385387,EF065541,EF538154,EU667476,EF210959,AY914845,EU385365,EF065540,EF538150,EU667509,EF210962,EF420897,EU385363,FJ789803,EF108401,EU667519,EF210939,AY914844,EU841322,FJ697137,EF028716,EU667520,EF210938,EF420880,EU385366,FJ697118,DQ915899,EU667498,EF210936,EF420898,EU385361,FJ697117,AB259333,EU667497,EF210935,EF420881,AF318919,FJ697096,AB259332,EU667529,EF210934,EF420909,EU385390,FJ697095,AB259331,EU667528,EF210974,EF420888,EU385383,FJ697136,AB259348,EU667467,EF210950,EF420896,EU385378,FJ697128,AB259347,EU667488,EF210940,EF420878,EU385357,FJ697127,AB259346,EU667465,AM690706,EF420900,EU385349,FJ697093,AB259345,EU667464,AM690703,EF420899,EU385339,FJ697122,AB259344,EU667463,AM690716,EF420884,EU385335,FJ697121,AB259343,EU667525,AM690730,EF420890,EU385354,FJ697148,AB259342,EU667501,AM690707,EF420906,AF318909,FJ697147,AB259341,EU667487,AM690702,EF420891,EU749318,FJ697097,AB259340,EU667470,AM690712,EF420895,EU749317,FJ697094,AB259339,EU667483,AM690736,EF091612,EU749315,FJ697150,AB259338,EU667522,AM690726,AY155663,EU337048,FJ697149,AB259337,EU667518,AM690723,AY155660,EU337056,FJ697124,AB259336,EU667516,AM690711,AY155658,EU749314,FJ697123,AB259335,EU667513,AM690708,AY155665,EU749313,FJ697140,AB259334,EU667510,AM690704,AY155656,EU749312,FJ697139,L48118,EU667506,AM690729,AY155650,EU749310,FJ697092,DQ451813,EF538208,AM690714,AY155648,AJ633140,FJ697091,DQ451815,AB355523,AM690728,FM994953,EU749321,FJ697090,DQ451809,AB355528,AM690722,FM994952,AJ633237,FJ697144,DQ451791,AB355499,AM690721,FM994951,EU749415,FJ697157,DQ451790,AB355498,AM690720,FM994950,EU749414,FJ697156,DQ451789,AB355497,AM690725,FM994949,AF151488,FJ697113,DQ451764,AB355496,AM690724,FM994948,EU749441,FJ697112,DQ451763,AB355495,FM177837,FM994947,EU749440,FJ697120,DQ451762,AB355494,AM690705,FM994954,EU749439,FJ697119,DQ451758,AB355493,AM690713,EF211002,GU135117,FJ697087,DQ451756,AB355492,AM690735,EF420893,DQ840448,FJ697086,DQ451754,AB355491,AM690719,DQ383912,DQ840447,FJ697142,DQ451797,AB355490,AM690734,DQ383907,DQ508004,FJ697141,DQ451795,AB355489,AM690733,DQ383906,DQ508003,FJ697099,DQ451760,AB355488,AM690718,DQ383903,EU385403,FJ697098,DQ451781,AB355487,EF538392,DQ383895,EU385399,FJ697089,DQ451765,AB355486,EF538376,DQ661036,EU841350,FJ697088,DQ451775,AB355485,EF091599,EF538080,EU385350,FJ697126,DQ451772,AB355484,EF091588,EF538052,FJ395428,FJ697125,DQ451759,AB355483,EF091580,AY155664,EU385326,FJ697100,DQ451757,AB355482,DQ889630,EF538049,AF456789,FJ697083,DQ319113,AB355527,DQ889636,EF538048,AJ633132,FJ697082,AM114331,AB355524,DQ444737,EF538047,FJ395393,FJ697135,FJ980338,EF577321,DQ889640,AY155662,AF456786,FJ697134,AY953927,EF577318,DQ444735,AY155661,GU109304,FJ697115,AY929903,EF577314,DQ889643,EF538046,EU046561,FJ697114,AY929902,EF577307,DQ444734,AY155659,AF456792,FJ697130,AY929901,EF577291,DQ444733,AY155655,AY009463,FJ697129,AY929900,EF577283,DQ444732,AY155652,EU736105,FJ697146,AY929899,EF577280,DQ444716,AY155651,EU736104,FJ697145,AY929898,EF577279,DQ444729,AY155645,EU736103,FJ697152,AY929897,EF577278,DQ444728,AY155644,AM234911,FJ697151,AY929896,EF577276,DQ444726,AY155643,AM234896,FJ697111,AY929895,EF577275,DQ444725,EF538082,AM234887,FJ697110,AY929894,EF577271,DQ444724,EF538055,AM234870,FJ697116,AY929893,EF577274,DQ444723,EF538039,AM234861,FJ640037,AY929892,EF538414,DQ444722,EF538078,AM234854,FJ640036,AY929891,EF538403,DQ889632,AY155642,AM234889,FJ640035,AY929890,EF538402,DQ444721,EF538051,AM234903,FJ640034,AY929889,EF538400,EF420936,EF538029,AM234876,FJ640032,AY929888,EF538397,EF420923,AY155640,DQ006061,FJ640031,AY929887,EF538394,DQ383884,EF538026,DQ006070,FJ640030,AY929886,EF538391,DQ383883,EF538061,AB530950,FJ640029,AY929885,EF538389,EF133504,GU216559,AM234865,FJ640027,AY929884,EF538387,DQ383882,GU216558,AM234863,FJ640028,AY929882,EF538383,DQ383881,GU216557,AM234848,FJ640026,AY929881,EF538382,DQ383870,GU216556,EU841107,FJ640025,AB435135,EF538381,DQ383869,DQ383891,EU841111,FJ640038,AB435132,EF538380,DQ383868,FJ846089,AB530951,AB457379,AB435128,EF538374,DQ383867,FJ846088,DQ006062,AB457378,AB435118,EF538373,DQ383866,FJ846087,AB530962,AB457377,AB435114,EF538370,DQ383864,DQ383890,AY395546,AB457376,AB435112,EF538368,DQ383863,DQ006151,AB530979,AB457375,AB435110,EF538362,DQ383860,EU841285,AB530975,AB457373,AB435104,EF538361,DQ383859,EU841299,AM234849,AB457372,AB457349,EF538358,DQ383858,EU841298,AB530981,AB457370,AB457348,EF538356,DQ383855,EU841292,AB530966,AB457369,AB457347,EF538355,DQ383875,EU841293,DQ006063,AB457368,AB457346,EF538353,DQ383873,EU841289,AF119187,AB457367,AB457345,EF538352,DQ383872,EU841288,AB530973,AB457366,AB457344,EF538351,DQ383853,EU841291,EU841123,AB457365,AB457343,EF538348,EF091592,EU841279,AY874428,AB457363,AB457342,EF538347,AM690709,EU841280,AB530956,AB457362,AB457341,EF538345,AY914833,EU841278,AY395530,AB457361,AB457340,EF538344,AY914832,EU841281,AB530952,AB457359,AB457339,EF538343,AY914830,EU841274,DQ673291,AB457356,AB457337,EF538341,AY914828,EU841270,DQ006058,AB457355,AB457335,EF538337,AY914826,EU841268,DQ006057,AB457354,AB457334,EF538335,AY914821,EF091601,AB530977,AB457353,AB457333,EF538334,AY914817,EF211011,L13646,AB457352,AB457332,EF538332,AY914816,EU547794,AB530954,AB457350,AB457331,EF538330,AY914814,AM690597,AB436869,AB436878,AB457382,AB436885,AB436870,AB436882,AB436896,AB436877,AB457380,AB436884,AB436860,AB436871,AB436866,AB436875,AB436872,AB436883,AB436889,AB436880,AB436864
