# Supplementary material for: Evaluating the feasibility of using candidate DNA barcodes in discriminating species of the large Asteraceae family
Source: BMC Evol Biol. 2010 Oct 26;10:324. doi: 10.1186/1471-2148-10-324 (PMC3087544; doi:10.1186/1471-2148-10-324)
Supplement: Additional file 5 — Accession numbers of ITS2 sequences used in dataset 2. For each samples used in dataset 2, the accession numbers in GenBank are shown. [file 1471-2148-10-324-S5.DOC]

### Additional file 5 – Accession numbers of ITS2 sequences used in dataset 2

AB064273,AB064275,AB064279,AB064283,AB118111,AB118118,AB118120,AB118124,AB118127,AB118128,AB118131,AB118132,AB118139,AB196595-AB196613,AB212735,AB245093,AB250266,AB250268,AB250269,AB250271,AB250272,AB250274,AB250278,AB250281,AB250283,AB259321,AB259322,AB259325,AB259326,AB259328,AB259331,AB259333,AB259336,AB259339,AB259340,AB259342,AB259346,AB259348,AB259350,AB284129,AB299047,AB355482,AB355483,AB355485,AB355489,AB355492,AB355495,AB355498,AB355499,AB355501,AB355502,AB355506,AB355508,AB355510,AB355512,AB355523-AB355528,AB359077,AB375311,AB435096-AB435100,AB435102-AB435151,AB435375,AB457234-AB457278,AB457280,AB457281,AB457282,AB457285,AB457287-AB457330,AB523364,AB523365,AF046951-AF046958,AF047900,AF047901,AF047902,AF047904-AF047927,AF047929-AF047957,AF047959-AF047973,AF061882-AF061914,AF116239,AF118478-AF118545,AF118914,AF118915,AF118916,AF118917,AF118918,AF118919,AF165829-AF165847,AF218884,AF229259-AF229272,AF229276-AF229323,AF251567-AF251598,AF251600,AF251602-AF251624,AF257785-AF257788,AF314591,AF314592,AF314593,AF314594,AF314595,AF314596,AF314600-AF314604,AF315080,AF330088-AF330102,AF386493-AF386503,AF391560,AF391562,AF391563,AF391564,AF391566,AF391568,AF391571-AF391588,AF391590,AF391593,AF391595-AF391622,AF412838,AF412839,AF412840,AF412843,AF412844,AF412845,AF412846,AF412849,AF412850,AF412851,AF412852,AF412854,AF412855,AF412856,AF412857,AF412859,AF412861-AF412877,AF412879-AF412887,AF422109-AF422115,AF422117-AF422137,AF422139,AF422140,AF429846,AF429856,AF429859,AF429860,AF429872,AF429873,AF429876,AF429878,AF430792,AF430793,AF430795,AF430796,AF430798-AF430803,AF437850,AF437852,AF459922-AF459925,AF459927-AF459935,AF459937,AF459938,AF459940,AF459941,AF459943-AF459972,AF465844-AF465852,AF465854-AF465908,AF490465-AF490585,AF492656,AF492657,AF492658,AF493994-AF494011,AF501626-AF501629,AF528433,AF528435-AF528442,AF528445-AF528466,AF528470,AF528475,AF528476,AF528484,AF528486-AF528491,AF528493,AF528494,AF528495,AF539922-AF539957,AF540013,AJ297261,AJ400777-AJ400808,AJ400810,AJ400811,AJ400813,AJ404744,AJ563924,AJ831528-AJ831538,AM086626,AM114278-AM114312,AM114313-AM114332,AM117044-AM117058,AM269938-AM269946,AM398844-AM398848,AM398850,AM398851,AM398852,AM398855-AM398861,AM398864,AM398865,AM398866,AM398868-AM398890,AM398892,AM398893,AM398894,AM398896-AM398903,AM398905-AM398922,AM398924-AM398927,AM398929,AM493990-AM493999,AM774421,AM774426,AM774427,AM774429,AM774430,AM774445,AM774447,AM774449-AM774453,AM774458,AM774461-AM774465,AM774467,AM774469-AM774473,AM932283,AY017351-AY017367,AY046932-AY046942,AY117440-AY117472,AY155603,AY155604,AY155605,AY155608-AY155613,AY169241,AY169242,AY226793,AY226795,AY275655,AY275656,AY327531,AY327537,AY327538,AY458588,AY548198-AY548201,AY548210,AY548211,AY554092-AY554096,AY554098-AY554114,AY576809,AY576815,AY576831,AY576843,AY576844,AY576848,AY576853,AY576854,AY576855,AY576860,AY576863,AY576865,AY576867,AY722010,AY826247,AY871316-AY871323,AY871334,AY871421,AY871470,AY871518,AY871568,AY871586,AY876260,AY876262,AY876263,AY876266,AY876268,AY876275,AY876276,AY876278,AY876280,AY876282,AY879152-AY879171,AY879173,AY879174,AY879179-AY879183,AY929881-AY953905,AY953907-AY953936,DQ005969,DQ005970,DQ005971,DQ005972,DQ005975,DQ005977,DQ005983,DQ101217,DQ122456,DQ122458,DQ122460,DQ122469,DQ122470,DQ122472-DQ122479,DQ122481-DQ122486,DQ122488-DQ122508,DQ122510,DQ122512,DQ122513,DQ122515,DQ122516,DQ122518,DQ122519,DQ122521,DQ122522,DQ122523,DQ122524,DQ122526,DQ122527,DQ122528,DQ122530,DQ122532,DQ122533,DQ122535-DQ122547,DQ159944,DQ198259,DQ272320-DQ272345,DQ310922-DQ310939,DQ310941-DQ310954,DQ319077-DQ319176,DQ322598,DQ322602,DQ322606,DQ322609,DQ322612,DQ322618,DQ355839-DQ355876,DQ355878-DQ355907,DQ355909-DQ355912,DQ355914,DQ355915,DQ355916,DQ355918,DQ355919,DQ355920,DQ383853-DQ383868,DQ383869-DQ383886,DQ391189-DQ391228,DQ414734,DQ444716-DQ444726,DQ444728-DQ444737,DQ451754,DQ451756-DQ451768,DQ451770,DQ451771,DQ451772,DQ451774-DQ451777,DQ451781,DQ451783,DQ451784,DQ451786,DQ451789,DQ451790,DQ451791,DQ451794-DQ451797,DQ451809,DQ451813,DQ451815,DQ451817,DQ451818,DQ451820-DQ451824,DQ478972-DQ478997,DQ478999-DQ479041,DQ479099-DQ479107,DQ479110,DQ479111,DQ479113-DQ479120,DQ629019-DQ629024,DQ813304,DQ826453,DQ862118-DQ862121,DQ889629-DQ889640,DQ889642,DQ889643,DQ889644,DQ915860-DQ915899,EF010530,EF055866,EF055867,EF065540,EF065541,EF065542,EF065544,EF065545,EF065546,EF065547,EF091575-EF091583,EF091585-EF091596,EF091598,EF091599,EF091600,EF103140,EF104922,EF107649-EF107658,EF108392-EF108405,EF114670,EF114672,EF123105,EF123106,EF133504,EF155744-EF155777,EF155779,EF155780,EF155782-EF155792,EF155794,EF155795,EF155796,EF155798-EF155813,EF155815,EF155816,EF155818-EF155827,EF155830,EF155831,EF177478,EF190030-EF190033,EF210928,EF210929,EF210930-EF210962,EF210964-EF210975,EF420915-EF420951,EF483945,EF483948,EF483949,EF483950,EF530219,EF530220,EF530221,EF530223,EF530225,EF530226,EF530229-EF530236,EF530238-EF530242,EF530247,EF530253-EF530262,EF538143,EF538144,EF538146,EF538148-EF538151,EF538154,EF538156,EF538158-EF538182,EF538185,EF538186,EF538189-EF538194,EF538196-EF538204,EF538206-EF538210,EF538212-EF538215,EF538218,EF538219,EF538220,EF538222,EF538223,EF538225-EF538229,EF538235-EF538244,EF538246-EF538251,EF538253,EF538254,EF538255,EF538257,EF538259,EF538260,EF538261,EF538263-EF538267,EF538272-EF538278,EF538280-EF538288,EF538291,EF538292,EF538294-EF538299,EF538301-EF538311,EF538315-EF538318,EF538320-EF538328,EF538330-EF538335,EF538337-EF538348,EF538351-EF538358,EF538360-EF538364,EF538366,EF538367,EF538368,EF538370,EF538372,EF538373,EF538374,EF538375,EF538376,EF538377,EF538378,EF538379,EF538380,EF538381,EF538382,EF538383,EF538386,EF538387,EF538389,EF538391,EF538392,EF538394,EF538396,EF538397,EF538399,EF538400,EF538402,EF538403,EF538405,EF538406,EF538407,EF538408,EF538411,EF538414,EF538416,EF543521,EF556320,EF556321,EF556322,EF556323,EF556328,EF556329,EF556330,EF556332,EF556335,EF556336,EF556337,EF556338,EF556339,EF556340,EF556341,EF556342,EF556343,EF556345,EF556346,EF556347,EF556348,EF556349,EF556350,EF556351,EF556353-EF556360,EF577271-EF577280,EF577282,EF577283,EF577284,EF577285,EF577287-EF577298,EF577300-EF577309,EF577312-EF577323,EF581385,EF627048,EF635446,EF635450,EF635458,EF635459,EF635481,EF635482,EF635483,EF635487,EF660536,EF660537,EF660540,EF660541,EF660542,EU007672-EU007680,EU007682,EU007683,EU007685,EU057986-EU169118,EU179212-EU179216,EU200184-EU200188,EU200215-EU200234,EU239682-EU239686,EU257418,EU257419,EU257420,EU257421,EU257423,EU257424,EU257425,EU257426,EU257427,EU257428,EU257429,EU331099,EU331117-EU331122,EU331138,EU331140-EU331143,EU352241-EU352247,EU409915-EU409919,EU527192-EU527232,EU527235-EU527240,EU621367,EU637105-EU637146,EU637148,EU637149,EU637150,EU637152,EU637155-EU637174,EU637185-EU637213,EU637215-EU637241,EU637252-EU637272,EU637275,EU637276,EU637278-EU637312,EU637314,EU637315,EU637316,EU637319-EU637340,EU667462,EU667463,EU667464,EU667465,EU667466,EU667467,EU667468,EU667469,EU667470,EU667471,EU667472,EU667473,EU667474,EU667475,EU667476,EU667477,EU667478,EU667480,EU667482-EU667531,EU729341-EU729344,EU781138-EU781143,EU781145,EU781147,EU781149,EU781166,EU781213,EU781227,EU781250,EU781252,EU781253,EU781279,EU781281,EU781288,EU781295,EU781306,EU781318,EU781321,EU781390,EU781400,EU781402,EU781403-EU781419,EU781446,EU781448,EU781456-EU781464,EU785941,EU796891,EU812812-EU812817,EU841142,EU841145,EU841146,EU841147,EU841148,EU841149,EU841151,EU841152,EU841154,EU841157,EU841158,EU841161,EU841164,EU841167,EU841169,EU841171,EU841173,EU841174,EU841175,EU841176,EU841177,EU853462,EU853464,EU886843,EU979538,EU979539,EU979540,FJ449865,FJ449875,FJ449882,FJ449884,FJ457927-FJ457931,FJ457933,FJ457934,FJ457935,FJ457936,FJ457937,FJ457941,FJ457942,FJ457943,FJ457944,FJ457946,FJ457947,FJ459646-FJ459688,FJ459690-FJ459694,FJ459696,FJ528300,FJ528301,FJ528302,FJ539125,FJ539127,FJ539128,FJ639910-FJ639921,FJ639925,FJ639926,FJ639927,FJ639928,FJ639929,FJ639930,FJ639932,FJ639934,FJ639935,FJ639937,FJ639938,FJ639941,FJ639942,FJ639944,FJ639946,FJ639947,FJ639949-FJ639953,FJ639955,FJ639956,FJ639958-FJ639962,FJ639964,FJ639965,FJ639967,FJ639970,FJ696702,FJ696703,FJ696961-FJ696965,FJ696967-FJ697073,FJ789805,FJ789806,FJ861471-FJ861480,FJ861493,FJ861499,FJ861506,FJ861511,FJ969854,FJ969855,FJ980316-FJ980324,FJ980327-FJ980351,FJ980353-FJ980359,FM177823,FM177824,FM177826,FM177827,FM177829-FM177835,FM177837,FM177841-FM177847,FM177850,FM177851,FM177853-FM177862,GQ396673,GQ478106,U67094-U67099,U67101,U67102,U67104,U67106-U67109,U67110,U67111,U67112,U67114,U69703-U69709,U95277-U95280,U95282-U95288,U95290-U95295.
